# OsPPR939, a *nad5* splicing factor*,* is essential for plant growth and pollen development in rice

**DOI:** 10.1007/s00122-020-03742-6

**Published:** 2021-01-02

**Authors:** Peng Zheng, Yujun Liu, Xuejiao Liu, Yuqing Huang, Feng Sun, Wenyi Wang, Hao Chen, Mehmood Jan, Cuicui Zhang, Yue Yuan, Bao-Cai Tan, Hao Du, Jumin Tu

**Affiliations:** 1 Institute of Crop Science, Zhejiang University, Hangzhou, 310058 China; 2 Key Laboratory of Plant Development and Environmental Adaptation Biology, Ministry of Education, School of Life Sciences, Shandong University, Qingdao, 266237 China; 3 College of Life Science and Technology, Guangxi University, Nanning, 530004 China

## Abstract

**Key message:**

P-subfamily PPR protein OsPPR939, which can be phosphorylated by OsS6K1, regulates plant growth and pollen development by involving in the splicing of mitochondrial *nad5* introns 1, 2, and 3.

**Abstract:**

In land plants, pentatricopeptide repeat (PPR) proteins play key roles in mitochondrial group II intron splicing, but how these nucleus-encoded proteins are imported into mitochondria is unknown. To date, a few PPR proteins have been characterized in rice (*Oryza sativa*). Here, we demonstrate that the mitochondrion-localized P-subfamily PPR protein OsPPR939 is required for the splicing of *nad5* introns 1, 2, and 3 in rice. Complete knockout or partial disruption of OsPPR939 function resulted in different degrees of growth retardation and pollen sterility. The dramatically reduced splicing efficiency of these introns in *osppr939-4* and *osppr939-5* led to reduced mitochondrial complex I abundance and activity and enhanced expression of alternative respiratory pathway genes. Complementation with *OsPPR939* rescued the defective plant morphology of *osppr939-4* and restored its decreased splicing efficiency of *nad5* introns 1, 2, and 3. Therefore, OsPPR939 plays crucial roles in plant growth and pollen development by splicing mitochondrial *nad5* introns 1, 2, and 3. More importantly, the 12th amino acid Ser in the N-terminal targeting sequence of OsPPR939 is phosphorylated by OsS6K1, and truncated OsPPR939 with a non-phosphorylatable S12A mutation in its presequence could not be imported into mitochondria, suggesting that phosphorylation of this amino acid plays an important role in the mitochondrial import of OsPPR939. To our knowledge, the 12th residue Ser on OsPPR939 is the first experimentally proven phosphorylation site in PPR proteins. Our results provide a basis for investigating the regulatory mechanism of PPR proteins at the post-translational level.

**Electronic supplementary material:**

The online version of this article (10.1007/s00122-020-03742-6) contains supplementary material, which is available to authorized users.

## Introduction

Cytoplasmic male sterility (CMS), a maternally inherited trait characterized by the inability of a plant to produce viable pollen grains, is caused by incompatibility between mitochondrial and nuclear genes. CMS has been detected in nearly 200 flowering plant species (Bentolila et al. 2002). The sterile phenotype of CMS lines can be restored by nuclear genes called restorer-of-fertility genes (*Rf* genes), which encode highly diverse proteins. For example, *Rf2*, the first plant restorer gene isolated in maize CMS-T, encodes an aldehyde dehydrogenase (Cui et al. 1996). *Rf17* in rice CMS-CW (Chinese wild rice) encodes a mitochondrial sorting protein containing an acyl-carrier protein synthase-like domain (Fujii and Toriyama 2009). The candidate gene for *Rf1* in sugar beet (*Beta vulgaris*) CMS-Owen, *bvORF20*, encodes a putative peptidase of the M48 family (Hagihara et al. 2005). However, most plant *Rf* genes encode pentatricopeptide repeat (PPR) proteins.

Rf-related PPR proteins are commonly targeted to mitochondria and suppress male sterility by controlling the expression of CMS-associated genes at the transcriptional and translational levels. RF1A and RF1B, two mitochondrion-localized proteins in rice CMS-BT (BoroII), restore male fertility by prohibiting ORF79 production via endonucleolytic cleavage (RF1A) or degradation (RF1B) of dicistronic B-*atp6/orf79* mRNA (Wang et al. 2006). In rice CMS-WA (Wild Abortive), RF4 suppresses WA352-mediated male sterility by decreasing *WA352* mRNA levels (Tang et al. 2014). The PPR protein RF5 physically interacts with the glycine-rich protein GRP162 to cleave the CMS-associated *atp6-orfH79* transcript in rice CMS-HL (Honglian), thus restoring fertility (Hu et al. 2012). Other PPR proteins also act as fertility restorers, including Rf-PPR592 in petunia (Bentolila et al. 2002), Rfo in Kosena rapeseed (Giancola et al. 2003), Rfk1 in radish (Koizuka et al. 2003), and PPR13 in sorghum (Koizuka et al. 2003).

PPR proteins are a large group of nucleus-encoded RNA-binding proteins containing a degenerate 35-amino acid motif (Small and Peeters 2000). Most PPRs are predicted to localize to plastids and/or mitochondria (Lurin et al. 2004). Structurally, PPRs are divided into the P and PLS subfamilies. P-subfamily PPR proteins contain canonical PPR motifs (P motifs, 35 amino acids long), whereas PLS-subfamily proteins possess triplets of P, L (35–36 amino acids), and S (31 amino acids) motifs, sometimes with additional C-terminal domains (the E, E + , and DYW domains) (Lurin et al. 2004; Cheng et al. 2016).

PPR proteins regulate gene expression in organelles at the post-transcriptional level, from RNA editing, intron splicing, RNA stability, and cleavage to translation (Lurin et al. 2004). The PPRs in the PLS-subfamily primarily participate in RNA editing (Choury and Araya 2006; Barkan and Small 2014) by changing cytidine to uridine at specific sites. Many PPRs, including CRR4 (Kotera et al. 2005), OTP72 (Chateigner-Boutin et al. 2013), and EMP5 (Liu et al. 2013), have been reported to involve in RNA editing in *Arabidopsis thaliana* and maize. Unlike PLS-subfamily PPR proteins, P-subfamily PPRs have demonstrated roles in intron splicing (Huang et al. 2018; Sun et al. 2019), transcript stabilization (Wang et al. 2017; Zhang et al. 2017), cleavage, and translation (des Francs-Small and Small 2014; Haili et al. 2016).

Because mitochondrial RNA lost the ability to undergo self-splicing, it requires PPR proteins to act as splicing factors (Bonen 2008; Brown et al. 2014). To date, many P-subfamily PPR proteins involved in mitochondrial intron splicing have been reported. The loss-of-function of these PPR genes leads to distinct defective phenotypes, highlighting the important roles of PPR proteins in plant growth and development. For example, *Arabidopsis thaliana* SLOW GROWTH 3 (SLO3) is involved in the splicing of *NADH dehydrogenase subunit7* (*nad7*) intron 2. *slo3* mutants with defects in the splicing of this intron show delayed germination, retarded growth, and delayed development (Hsieh et al. 2015). ORGANELLE TRANSCRIPT PROCESSING 43 (OTP43) is required for the *trans*-splicing of *nad1* intron 1. An *Arabidopsis* mutant with a disrupted *OTP43* gene shows curled leaves and malformed seeds (de Longevialle et al. 2007). In maize, *Defective kernel 35* (*DEK35*) is essential for mitochondrial *nad4* intron 1 splicing. The *dek35* mutant exhibits impaired mitochondrial structure and delayed seed development (Chen et al. 2017). *Empty Pericarp8* (*Emp8*) functions in the *trans*-splicing of *nad1* intron 4 and the *cis*-splicing of *nad4* intron 1 and *nad2* intron 1. The *emp8* mutation is embryonic lethal, resulting in severely arrested embryo and endosperm development (Sun et al. 2018). Both PPR101 and PPR231 are required for the splicing of mitochondrial *nad5* introns 1 and 2, whereas PPR231 is also required for *nad5* intron 3 and *nad2* intron 3 splicing. Loss-of-function of PPR101 and PPR231 generates empty pericarp and small kernel phenotype, respectively (Yang et al. 2020).

Phosphorylation is a common post-translational modification that plays essential roles in many cellular signaling pathways and networks by altering the structures, localizations, and functions of proteins. The phosphorylation of the transit peptides of nucleus-encoded preproteins regulates the import of proteins into chloroplasts. For example, three plant-specific STY kinases can phosphorylate the transit peptide of the precursor of the small subunit of Rubisco (pSSU) (Lamberti et al. 2011a, b). Phosphorylated pSSU shows more import-competent than the unphosphorylated monomeric form by forming a guidance complex with heat shock protein 70 and 14-3-3 proteins (May and Soll 2000). The phosphorylation of presequences of nucleus-encoded mitochondrial preproteins might also participate in the import process (Law et al. 2015, 2018). The presequence of multiple organellar RNA editing factors 3 (MORF3) is phosphorylated by cytosolic STY kinases. After phosphorylation, the import of pMORF3 into mitochondria is impeded (Law et al. 2015).

Most P-subfamily PPR proteins implicated in intron splicing in the mitochondria reported to date are from *Arabidopsis* and maize. Although some PPRs have been characterized in rice, most of them are required for RNA editing of mitochondrial transcripts or RNA editing and intron splicing in chloroplasts (Toda et al. 2012; Tang et al. 2017; Xiao et al. 2018; Zhang et al. 2020). By contrast, only a few P-subfamily PPR proteins in rice were identified to function in mitochondrial intron splicing (Wu et al. 2019, 2020; Xue et al. 2019).

In this study, we characterized a P-subfamily PPR protein, OsPPR939, which is associated with the development of rice. OsPPR939 acted as a splicing factor required for the splicing of the mitochondrial *nad5* introns 1, 2, and 3. The loss-of-function of OsPPR939 seriously impaired the activities of the mitochondrial electron transport chain complexes and resulted in reduced plant height and pollen viability. We next demonstrated that the 12th residue Ser in the N-terminal sequence of OsPPR939 was phosphorylated by the kinase OsS6K1, which might be important for the import of OsPPR939 into mitochondria. Our results indicate that OsPPR939 plays crucial roles in the maintenance of mitochondrial function, plant growth, and pollen development and provide insight into the regulation of PPR proteins at the post-translational level.

## Materials and methods

### Plant materials and growth conditions

The *osppr939* mutants were generated using the CRISPR/Cas9 system. The CRISPR/Cas9 plasmids were constructed as described previously (Liu et al. 2017a). The 20-bp guide sequences (Table S1) within the target regions were selected, and their targeting specificity was analyzed using CRISPR-P 2.0 (Liu et al. 2017b). The *osppr939* mutants with mutations in MS1, 4, and 5 were in the Nipponbare (*Oryza sativa.* L. *Japonica*) background, and the *osppr939* mutants with mutations in MS2 and 3 were in the Zhonghua 11 (*Oryza sativa.* L. *Japonica*) background. Calli derived from mature seeds were used for *Agrobacterium*-mediated transformation. Genomic DNA was extracted from the transgenic lines by the cetyltrimethylammonium bromide (CTAB) method. The genomic region surrounding the CRISPR target sites of *osppr939* was amplified by PCR using specific primers (Table S2), and the segment was subjected to Sanger sequencing to identify mutants. Rice plants used in this study were grown in the paddy field and greenhouse in Zhejiang University under proper management.

The *tang2* mutant (SALK_003139) was a gift from Prof. Catherine Colas des Francs-Small (University of Western Australia). Both wild type and *tang2 Arabidopsis thaliana* mutants were in the Columbia-0 (Col-0) ecotype background. The plants were grown in soil at 22 °C under a 16-h light/8-h dark photoperiod.

### Bioinformatic analysis

The full-length amino acid sequences of OsPPR939 and its 15 homologs were retrieved by BLASTP analysis (http://www.ncbi.nlm.nih.gov/Blast.cgi). Multiple protein sequence alignments were performed using Clustal X. A phylogenetic tree was constructed using MEGA v.6.0 (http://www.megasoftware.net/index.html) based on the neighbor-joining method with the following parameters: p-distance model, pairwise deletion, and bootstrap (1000 replicates; random seed). Bootstrap support values for each node from 1000 replicates are shown next to the branches. The 30 N-terminal amino acids of OsPPR939 and its Clustal X-aligned counterparts from the 15 homologs were used to generate a sequence logo using WebLogo (http://weblogo.berkeley.edu/logo.cgi).

### RNA extraction, RT-PCR and qRT-PCR analysis

Total RNA was isolated from rice tissues using RNAiso Plus reagent (TaKaRa), including root, stem, leaf, callus, anther, pistil, palea, and lemma tissue and anther tissue at different stages (determined based on spikelet length). The quality and quantity of total RNA were analyzed using a NanoDrop 1000 spectrophotometer (Thermo Fisher Scientific, Waltham, MA). Approximately 2 µg of RNA per sample was used to synthesize cDNA with a PrimeScript RT reagent Kit with gDNA eraser (TaKaRa). Two microliters of a tenfold dilution of the cDNA solution were used as the template for RT-PCR*.* qRT-PCR was performed on a LightCycler 96 (Roche Diagnostics) using TaKaRa SYBR Premix Ex Taq II with a standard two-step protocol consisting of 95 °C for 30 s, followed by 40 cycles of 95 °C for 5 s and 60 °C for 30 s. PCRs were performed in three biological replicates, each with three technical replicates. *OsGAPDH* was used as an internal control. All primers used for (q)RT-PCR are listed in Table S2.

### Mitochondrial complex activity and immunoblotting assays

Crude mitochondria were isolated from rice calli as described previously (Cai et al. 2017). Mitochondrial proteins (130 µg) were solubilized in 1% N- dodecyl maltoside β-DM (Sigma, Santa Clara, CA, USA) and separated by 3–12.5% BN-PAGE. The gel was stained by Coomassie Brilliant Blue R-250 or subjected to an in-gel complex I activity assay as described (Meyer et al. 2009). Mitochondrial proteins and total proteins were used for immunoblot analysis. Total proteins were extracted from wild type and *osppr939-4* calli as follows: the calli were homogenized in liquid nitrogen using a mortar and pestle. Each 0.1 g sample of homogenized calli was combined with 200 µl extraction buffer (50 mM Tris–HCl, pH 7.5, 150 mM NaCl, 0.5% TritonX-100, protease inhibitor [Roche]) and centrifuged at 12,000 rpm at 4 °C for 10 min. The supernatant was transferred to a new tube, and protein content was determined using an Enhanced BCA Protein Assay Kit (Beyotime, Shanghai, China). One-third volume of 4 × Laemmli buffer (250 mM Tris–HCl, pH 6.8, 8% SDS, 40% glycerol, 4% β-mercaptoethanol, 0.01% bromophenol blue) was mixed with the supernatant. The mixture was denatured at 95–100 °C for 10 min. The mitochondrial proteins (12 µg) or total proteins (10 µg) were separated by sodium dodecyl sulfate-PAGE (SDS-PAGE), transferred onto a polyvinylidene difluoride (PVDF) membrane (Bio-Rad), and incubated with various primary antibodies against *Arabidopsis* Nad4 and Nad7 (PhytoAB; PHY0511S and PHY0513S, respectively), wheat Nad9, maize Cyt*c*_*1*_, ATPase-α subunit, *Arabidopsis* Cox2 (Xiu et al. 2016), or rice OsGAPDH (Beijing Protein Innovation, Beijing, China). Detection was carried out using ECL Western Blotting Detection Reagents (Bio-Rad).

### Measuring ATP content and examining pollen viability

The ATP content of calli generated from mature rice seeds was measured with a Luciferin-luciferase ATP Assay Kit (Beyotime, Shanghai, China). To analyse pollen viability, mature anthers from wild type and mutant plants were immersed into 1% (w/v) iodine and potassium iodide (I_2_–KI) solution at room temperature with three biological replicates. The stained pollen grains were visualized and photographed under a Leica DMIRB fluorescence microscope.

### Scanning electron microscopy analysis

For SEM analysis, mature anthers collected from wild type (Nipponbare), and *osppr939-4* plants were fixed in 2.5% (v/v) glutaraldehyde and dehydrated through an alcohol gradient (70%, 80%, 95%, and 100%). The samples were critical point dried using a Leica EMCPD300 critical point drier and observed under a scanning electron microscope (Hitachi TM-1000, Tokyo, Japan) at an accelerating voltage of 15 kV.

### In vitro kinase assay

For the in vitro kinase assay, different HA-fused kinases were separately expressed in 4 × 10^5^ rice protoplasts and purified as follow: The protoplasts were lysed in 1 mL immunoprecipitation buffer containing 50 mM Tris–HCl pH 7.5, 150 mM NaCl, 5 mM EDTA, 1 mM DTT, 2 mM NaF, 2 mM Na_3_VO_4_, 1% Triton X-100, and 1 × protease inhibitor cocktail (Complete mini, Roche). The protein extracts were incubated with 2 μg anti-HA antibody (Sigma, F1804) at 4 °C for 2 h and an additional 1 h with protein G Sepharose beads (GE Healthcare). The immunoprecipitated kinase protein was washed three times with immunoprecipitation buffer and once with kinase buffer (25 mM Tris–HCl, pH 8.0, 100 mM NaCl, 10 mM MgCl_2_, 5 mM CaCl_2_, and 1 mM DTT). The substrates OsPPR939N^1−28^-GST and OsPPR939N^1−28^S12A-GST, OsPPR939N^1−28^S12T-GST, and OsPPR939N^1−28^S12R-GST were expressed in *E. coli* strain BL21 cells and purified. The in vitro kinase assay was performed by incubating purified kinase and 0.2 μg substrate in reaction buffer containing 25 mM Tris–HCl, pH 8.0, 100 mM NaCl, 10 mM MgCl_2_, 5 mM CaCl_2_, 0.1 μM ATP, and 6 µCi [γ-^32^P] ATP (Perkin-Elmer) at 30 °C for 30 min. The reaction was stopped by adding 15 μL of 6 × SDS-PAGE loading buffer. After separation the proteins on a 15% SDS-PAGE gel, protein kinase activity was detected on the dried gel using a Typhoon imaging system (GE Healthcare Life Sciences).

### Subcellular localization of OsPPR939

To verify the subcellular localization of OsPPR939, the 840-bp *OsPPR939* gene sequence beginning at the start codon was amplified by PCR from wild type Nipponbare and cloned into pENTR/D-TOPO (Invitrogen, USA). The fusion construct was introduced into the binary vector pGWB5, which contains the cauliflower mosaic virus 35S promoter, by Gateway site-specific recombination (Invitrogen). For stable expression in *Arabidopsis*, the pGWB5-*OsPPR939N*^1−280^:*GFP* construct was transformed into wild type *Arabidopsis* plants by the floral dip method. Protoplasts extracted from transgenic *Arabidopsis* plants expressing OsPPR939N^1−280^:GFP fusion protein was stained with MitoTracker Red. To further validate the contribution of the phosphorylated N-terminus to the subcellular localization of OsPPR939, we cloned gene fragments expressing OsPPR939N^1−280^, the truncated derivative OsPPR939N^29−280^, and three point mutation derivatives (OsPPR939N^1−280^S12A, OsPPR939N^1−280^S12T, and OsPPR939N^1−280^S12R) into the pM999-GFP vector. The primers are listed in Table S2. The plasmids were transformed into rice protoplasts isolated from 10-day-old etiolated Zhonghua 11 seedlings with the mitochondrial marker *F1-ATPase-γ:RFP* (Jin et al. 2003) by polyethylene glycol-mediated transformation. The GFP and RFP signals were detected under a Zeiss LSM780 confocal microscope.

### Complementation analysis

For functional complementation, wild type (Nipponbare) genomic DNA was used as a template to amplify a 7191-bp genomic DNA fragment of *OsPPR939* containing the 4371-bp upstream region and 2820-bp coding region. The amplified fragment was subcloned into the pCAMBIA1300 vector by In-Fusion cloning (TaKaRa Bio, Japan). The resulting plasmid containing *OsPPR930pro-OsPPR939 gDNA* was transformed into *Agrobacterium tumefaciens* EHA105 and used for floral dip transformation of *tang2* plants and callus transformation of *osppr939-4* plants. Transformed plants were selected on hygromycin, and independent transgenic lines were identified by PCR analysis.

#### RNA in situ hybridization

RNA in situ hybridization was performed as described previously (Liu et al. 2017a). Gene-specific *OsPPR939* cDNA fragments generated by PCR were used to prepare antisense and sense probes under the control of the T7 and SP6 promoter with RNA polymerase using a DIG RNA Labeling Kit (Roche). PCR primers are listed in Supplemental Table S2.

## Results

### Generation of *osppr939* mutants by CRISPR/Cas9-mediated genome editing

**Fig. 1:**
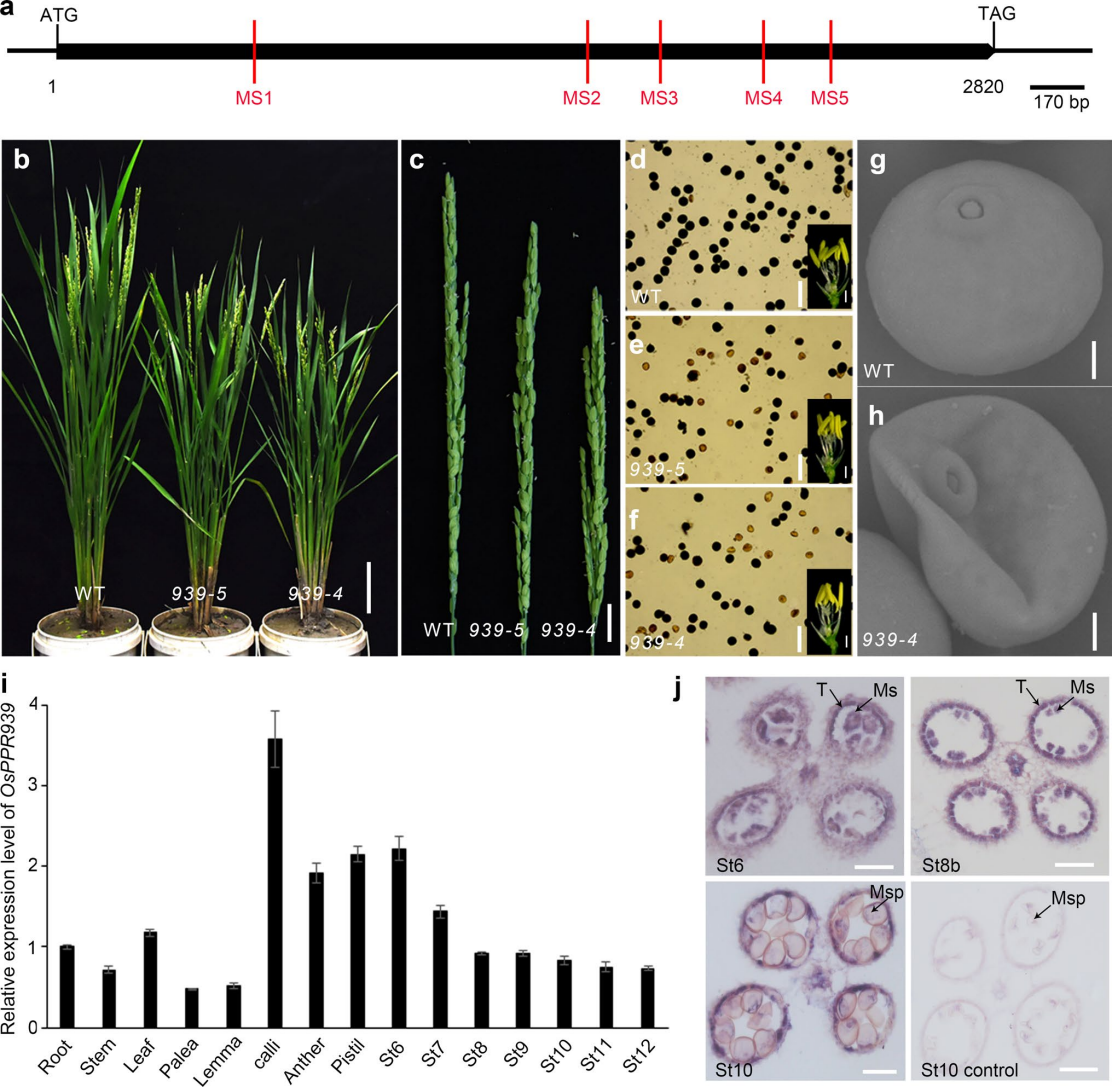
Phenotypic characterization of *osppr939* mutants and expression analysis of *OsPPR939.* **a** Schematic diagram of the intronless gene *OsPPR939*. Red line indicates the location of each mutation site in *osppr939*. **b** Wild type and mutant plants at the heading stage. Bars = 10 cm. **c** Rice panicles at the heading stage. Bars = 1 cm. **d**–**f** I_2_-KI staining of pollen grains from the wild type, *osppr939-5* and *osppr939-4*, respectively. Insets show the spikelets without the lemma and palea. Bars = 100 μm. **g** and **h** Higher magnification views of SEM images showing a single pollen grain in the wild type and *osppr939-4*. Bars = 10 μm. **i** Expression analysis of *OsPPR939* by qRT-PCR. RNAs were extracted from the root, stem, leaf, palea, lemma, calli, anther, pistil, and spikelets from stage 6 to stage 12. Error bars indicate the SE based on three biological replicates. *OsGAPDH* was used as an internal control. St, stage. **j** In situ hybridization analysis of *OsPPR939* in wild type anthers. Anthers at stage 6, stage 8b, and stage 10 with *OsPPR939* antisense probe. Anther at stage 10 with the sense probe served as a negative control. T, tapetum; Ms, microsporocyte; Msp, microspores. Bars = 10 μm

We previously identified OsPPR939 in a yeast two-hybrid screen in which five proteins including OsPPR939 were confirmed to interact with OsjBTF3 and shown to be involved in the *OsjBTF3*-regulated pathway controlling plant growth and pollen development in rice (Wang et al. 2012). Here, to investigate the function of *OsPPR939*, we selected five mutation sites (MS1–MS5) in different regions of this gene to generate *osppr939* mutants using the CRISPR/Cas9 system (Fig. 1a). CRISPR/Cas9 constructs expressing guide RNA sequences targeted to the five mutation sites were independently transformed into rice calli (MS1, 4, and 5 in Nipponbare and MS2 and 3 in Zhonghua 11). Sequencing of the DNA covering the mutation sites amplified from *T*_0_ transgenic plants confirmed the existence of independent mutations at each mutation site, namely MS1 to MS5 (Table S1).

Surprisingly, all *T*_0_ plants with mutations at MS1, MS2, and MS3 were heterozygous, whereas *T*_0_ plants with mutations at MS4 and MS5 were either homozygous or heterozygous. Perhaps MS4 and MS5 are located at the C-terminus, allowing the mutated OsPPR939 to retain part of its function and thereby ensuring homozygous mutant formation. To generate a complete loss-of-function mutant, we performed segregation analysis using *osppr939-1, -2,* and *-3* plants heterozygous for MS1, 2, and 3, respectively. The progeny of both *osppr939-1* and *osppr939-2* showed 1:1 segregation of wild type (WT/WT) to heterozygous (WT/−1 bp) plants. The segregation ratio of WT/WT:WT/−1 bp in *osppr939-3* was 1:2 instead of 1:1, and no homozygous mutant was isolated from the offspring of these three heterozygotes (Table 1).

**Table 1:** Segregation analysis of *osppr939* heterozygous mutants

Transgenic *T*_1_ lines	Genotype	Segregation of *T*_2_ plants			Segregation ratio WT/WT:WT/−1 bp	χ^2^	*p* value
		WT/WT	WT/−1 bp	−1 bp/−1 bp			
*osppr939-1*	WT/−1 bp	79	78	0	1:1	0.06	0.94
*osppr939-2*	WT/−1 bp	68	60	0	1:1	0.50	0.48
*osppr939-3*	WT/−1 bp	49	92	0	1:2	0.13	0.72

We reasoned that perhaps pollen with the *osppr939-1* or *-2* genotype was nonviable, while pollen with the *osppr939-3* genotype survived and completed double fertilization of oocytes, but the resulting homozygous *osppr939* zygotes died at an early stage. To confirm our hypothesis, we performed reciprocal crosses using the *osppr939-1* heterozygote (WT/−1 bp) as the female parent and the wild type (WT/WT) as the male parent or vice versa. The progeny from the forward cross included 50 WT/WT and 57 WT/−1 bp plants, corresponding to a 1:1 segregation ratio (*χ*^2^ = 0.46, *P* > 0.05). By contrast, all progeny from the reverse cross were WT/WT (Table 2). These results demonstrate that *OsPPR939* is crucial for pollen development in rice and that its mutation results in non-viability of male gametes but does not affect female gamete development.

**Table 2:** Reciprocal crosses between *osppr939-1* heterozygous mutants and the wild type

Parent		Progeny	
Female (♀)	Male (♂)	WT/−1 bp	WT/WT
*osppr939-1* (WT/−1 bp)	Wild type (WT/WT)	57	50
Wild type (WT/WT)	*osppr939-1* (WT/−1 bp)	0	71

### Phenotypic analysis of *osppr939* mutants

Because *osppr939-4* and *osppr939-5*, which were created by CRISPR/Cas9 at the MS4 and MS5 loci, respectively, were homozygous mutants with similar phenotypes, we selected these mutants for subsequent experiments. *osppr939-4* contains a frame-shift due to a one base insertion, resulting in the termination of protein translation at the 17th PPR repeat (Fig. S1a, c). Similarly, *osppr939-5* contains a frame-shift induced by a two base pair deletion, resulting in the termination of protein translation at the 19th PPR repeat (Fig. S1b, c). When we backcrossed *osppr939-4* with the wild type, all F_1_ progeny displayed the wild type phenotype, and the F_2_ progeny showed a 3:1 segregation ratio of wild type to mutant plants (98:33, *χ*^2^ = 0.0025, *P* > 0.05), suggesting that this mutation is monofactorial recessive. Both *osppr939-4* and *osppr939-5* homozygous plants showed mildly retarded growth at the seedling stage (Fig. S2a, b), significantly reduced plant height, and shorter panicles than the wild type at the heading stage (Fig. 1b, c, Fig. S2c). However, these defects were more severe in *osppr939-4* than in *osppr939-5*. The pollen grains of *osppr939-4* and *osppr939-5* were partially sterile, as revealed by iodine potassium iodide (I_2_-KI) staining (Fig. 1d–f, Fig. S2d), although their floral organs developed normally (insets in Fig. 1d–f).

This phenotype was further confirmed by scanning electron microscopy. Unlike the plump and regular wild type pollen grains, approximately half of the pollen grains from *osppr939-4* were severely collapsed and shrunken, while the remaining pollen grains resembled those of the wild type (Fig. 1g, h). As a result, many empty grains appeared in the spikes of both mutants (Fig. S3a), and the seed setting rates were ~ 50%, i.e., significantly lower than the 82% seed setting rate of the wild type (Fig. S3b). Reverse-transcription (RT)-PCR analyses revealed that the steady-state levels of *OsPRR939* in the mutants were indistinguishable from those of the wild type (Fig. S4), indicating that the deletion or insertion of a few nucleotides did not significantly affect the stability of these transcripts, which is consistent with a previous report (Hsieh et al. 2015).

### *OsPPR939* encodes a mitochondrion-localized P-subfamily PPR protein

**Fig. 2:**
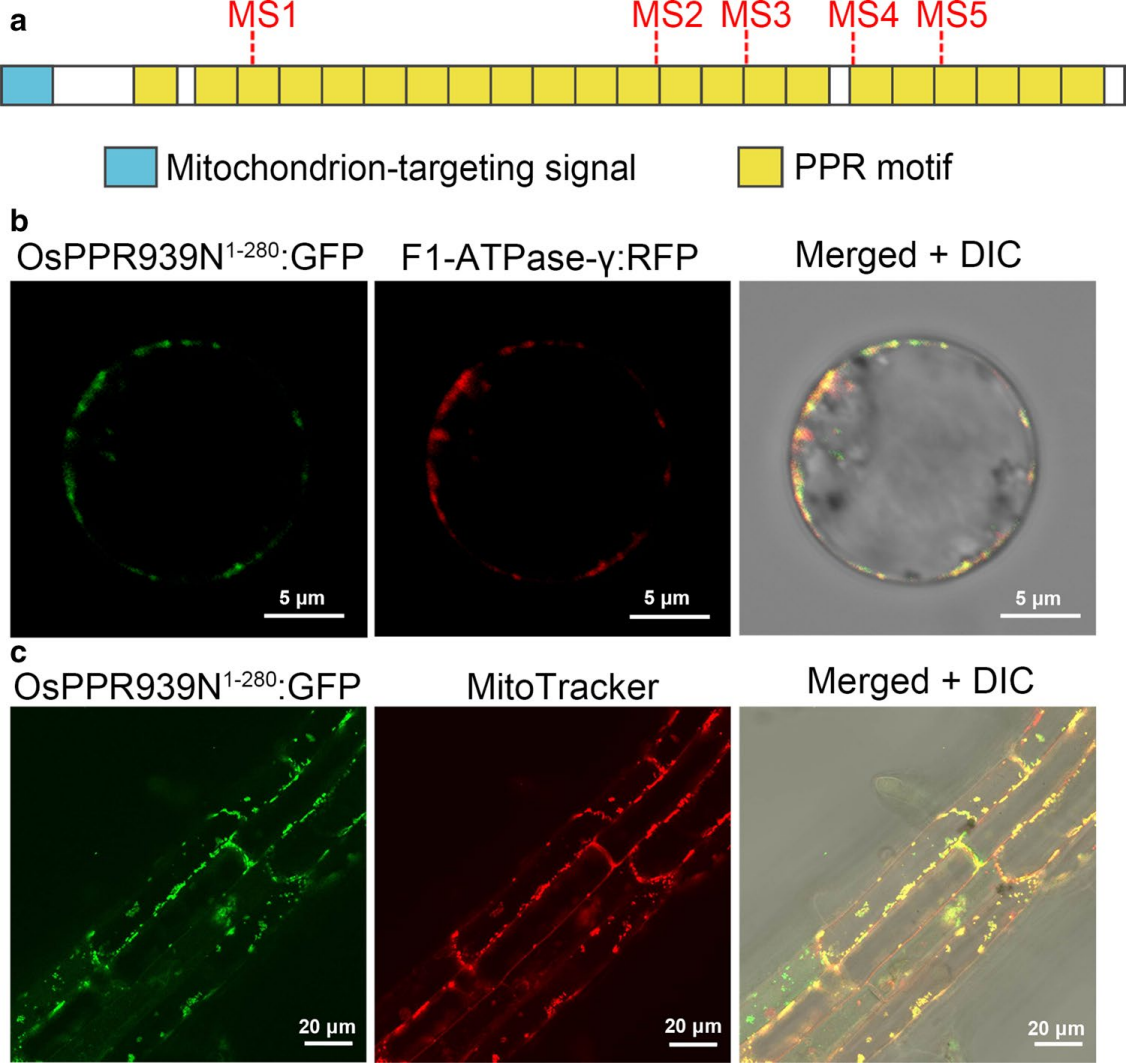
Schematic of OsPPR939 and subcellular localization of GFP-tagged OsPPR939N^1−280^. **a** OsPPR939 protein contains 22 PPR motifs and a mitochondrion-targeting peptide at the N-terminus. **b** Subcellular localization analysis of OsPPR939N^1−280^:GFP by coexpression with F1-ATPase-γ:RFP (mitochondrial marker) in rice protoplasts. DIC, differential interference contrast*.* Bars = 5 μm. **c** Subcellular localization of OsPPR939N^1−280^:GFP in the roots of stable transgenic *Arabidopsis.* Mitochondria were stained with MitoTracker Red. DIC, differential interference contrast*.* Bars = 20 μm

Sequence analysis revealed that full-length *OsPPR939* cDNA contains a single exon and encodes a protein of 939 amino acids. Database searches (TPRpred: https://toolkit.tuebingen.mpg.de/tools/tprpred) showed that OsPPR939 contains 22 PPR motifs and no E, E + , or DYW domains at its C-terminus, indicating that OsPPR939 is a P-subfamily PPR protein (Fig. 2a).

Most PPR proteins are targeted to mitochondria and/or plastids (Lurin et al. 2004). Based on analysis using the TargetP algorithm (http://www.cbs.dtu.dk/services/TargetP/) and Predotar (https://urgi.versailles.inra.fr/predotar/predotar.html), OsPPR939 was predicted to contain a mitochondrion-targeting peptide (MT) at its N-terminus (1–28 a.a.) (Fig. 2a), suggesting that it localizes to mitochondria. To experimentally determine the subcellular localization of OsPPR939, we expressed full-length OsPPR939 fused with green fluorescence protein (GFP) driven by the 35S promoter in wild type rice. However, we did not observe any GFP signals from seedling roots in any positive transgenic line (as identified by PCR). Thus, we fused the 280 N-terminal amino acids containing the predicted mitochondrion-targeting peptide with GFP. We cotransformed rice protoplasts with the OsPPR939N^1−280^:GFP construct and the mitochondrial marker F1-ATPase-γ:RFP (Jin et al. 2003). Both GFP and RFP signals were detected by confocal microscopy. The green fluorescence colocalized with the red fluorescence (Fig. 2b). We also introduced OsPPR939N^1−280^:GFP into wild type *Arabidopsis* Columbia-0 (Col-0) plants via the floral dip method and stained roots, leaves, and protoplasts from these plants with MitoTracker Red to label their mitochondria. In all tissues examined, the GFP signals of OsPPR939 N^1−280^:GFP overlapped with the RFP signals from MitoTracker Red (Fig. 2c, Fig. S5). These results suggest that OsPPR939 localizes to the mitochondria in rice.

### Phylogenetic analysis of OsPPR939

To explore the evolution of OsPPR939, we performed a BLASTP search of the National Center for Biotechnology Information database (NCBI, http://www.ncbi.nlm.nih.gov/) using the full-length OsPPR939 sequence as a query. Homologs of OsPPR939 were only detected in angiosperm species, and no paralog of OsPPR939 is present in rice. Phylogenetic analysis of the 15 homologs retrieved from 15 different species, and the full-length OsPPR939 clearly divided the 16 proteins into two clusters (Fig. S6a). In the monocotyledon cluster, OsPPR939 shares sequence identities of 65.39%, 71.40%, 73.97%, 69.35%, 68.39%, and 70.82% with its orthologs in *Oryza brachyantha*, *Brachypodium distachyon*, *Aegilops tauschii*, *Setaria italica*, *Zea mays*, and *Sorghum bicolor*, respectively, although the functions of these proteins are largely unknown. Among the dicotyledon species, *Arabidopsis* TANG2 (annotated as NP_173362.2) is the most closely related homolog; this protein is essential for mitochondrial intron splicing (des Francs-Small et al. 2014). Although TANG2 only shares 37.24% amino acid sequence identity with OsPPR939, the locations of most PPR repeats are highly conserved in these two proteins, except for the last two repeats, which only exist in OsPPR939 (Fig. S6b). Together, these results indicate that OsPPR939 is a typical P-subfamily PPR protein and may also be involved in mitochondrial intron splicing in rice.

### Expression analysis of *OsPPR939*

We examined the expression pattern of *OsPPR939* by quantitative real-time (qRT)-PCR using total RNA extracted from vegetative and reproductive organs of wild type plants. *OsPPR939* was ubiquitously expressed in all tissues examined, with peak expression in calli and a relatively high expression level in spikelets at stage 6 (St6), which gradually decreased during later stages of development. *OsPPR939* was also highly expressed in anther and pistil tissue and relatively weakly expressed in root, stem, leaf, palea, and lemma tissue (Fig. 1i).

To investigate the spatial and temporal expression patterns of *OsPPR939* during pollen development, we performed RNA in situ hybridization using wild type anther sections. Strong hybridization signals were detected in both the tapetum and male meiocytes at stages 6, 8b, and 10, while only background levels of signal were detected with the sense probe (Fig. 1j). Since the tapetum plays a crucial role in regulating pollen formation, these data suggest that OsPPR939 is involved in pollen development.

### OsPPR939 functions in mitochondrial *nad5* intron splicing

**Fig. 3:**
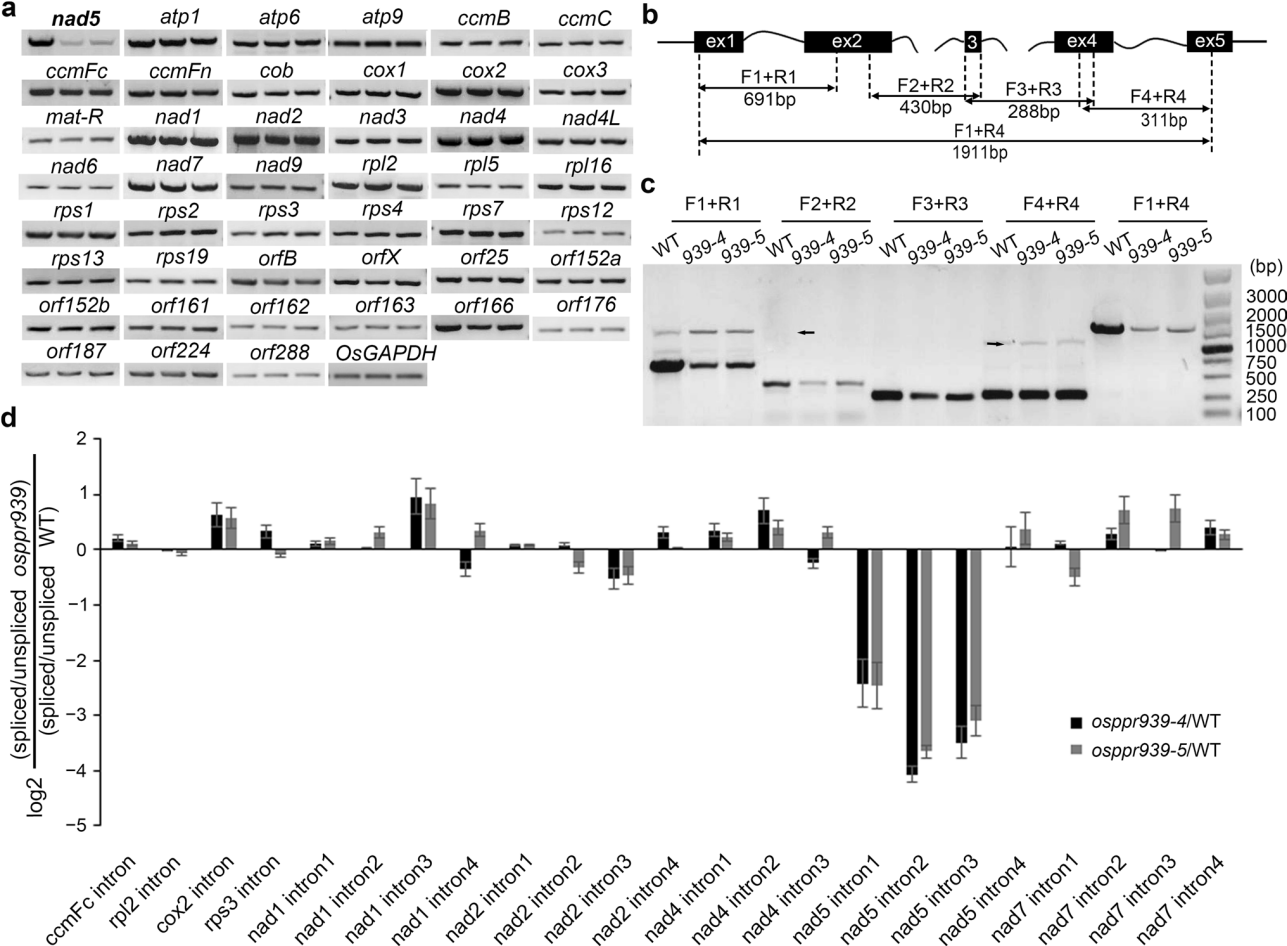
The *osppr939* mutants show reduced mature mitochondrial *nad5* transcript levels and deficient splicing of *nad5* introns 1, 2, and 3. **a** RT-PCR analysis of the transcript levels of 45 mitochondria-encoded genes in the wild type (WT; left lane), *osppr939-4* (middle lane), and *osppr939-5* (right lane). Total RNA was isolated from the leaves of plants at the flowering stage. *OsGAPDH* was used as an internal control. **b** Structure of the rice mitochondrial *nad5* gene. Introns 1 and 4 are *cis*-spliced introns and introns 2 and 3 are *trans*-spliced introns. The expected amplification products of different primer pairs are indicated. **c** RT-PCR analysis of the intron splicing efficiency of *nad5* in wild type and *osppr939* plants. Solid arrows correspond to unspliced fragments. **d** qRT-PCR analysis of the splicing efficiency of all 23 group II introns in *osppr939-4*, *osppr939-5*, and wild type plants. Error bars indicate SEs based on three biological replicates

PPR proteins are RNA-binding proteins that function in RNA metabolism, including RNA splicing (Barkan and Small 2014). Many PPR proteins, especially members of the P-subfamily, participate in organellar RNA intron splicing. To verify whether this was the case for OsPPR939, we measured the levels of 45 mitochondrion-encoded transcripts in wild type and *osppr939* plants by RT-PCR analysis. We extracted total RNA from wild type and mutant leaves and performed RT-PCR using specific primers designed to amplify the transcript of each mitochondrial gene (Table S2). *nad5* transcript levels were dramatically reduced in the mutants, whereas the levels of most transcripts were indistinguishable between the wild type and *osppr939* (Fig. 3a). These results suggest that OsPPR939 might be essential for the splicing or stability of *nad5* transcript.

The mitochondrial gene *nad5* encodes a subunit of the mitochondrial respiratory complex I (NADH:ubiquinone oxidoreductase) (Meyer 2012). Rice *nad5* consists of five coding segments, which are located in a wide region of the mitochondrial genome (Notsu et al. 2002). The formation of the mature *nad5* transcript requires two *cis*-splicing events of intron 1 and 4 as well as two *trans*-splicing events of introns 2 and 3 (Fig. 3b) (Knoop et al. 1991). To examine whether the weak accumulation of mature *nad5* transcript in the *osppr939* mutants was caused by a deficiency in intron splicing, we examined the splicing of *nad5* precursor transcript. Four specific primer pairs were designed to amplify fragments containing each of the four introns in the *nad5* transcript (Fig. 3b). In both *osppr939-4* and *osppr939-5*, the levels of the spliced fragments joining exon 1 and exon 2 were much lower, and the levels of the unspliced fragments containing *nad5* intron 1 were higher than those in the wild type when we used the primer pair F1 + R1 for amplification (Fig. 3c). The levels of intron 2 and intron 3 spliced fragments, which were amplified using primer pairs F2 + R2 and F3 + R3, respectively, were also lower in the mutants than the wild type. However, intron 2 and intron 3 unspliced transcripts were not detected in wild type or mutant plants due to the failure to perform PCR amplification of such long fragments containing both intron 2 and intron 3 (Fig. 3c). However, there was no difference in the abundance of intron 4-spliced and intron 4-unspliced fragments between wild type and the mutants using primer pair F4 + R4 (Fig. 3c). Similar results were obtained in rice calli (Fig. S7).

To further investigate the changes in splicing in the *osppr939* mutants, we designed specific primers for qRT-PCR to detect the splicing efficiency of 23 mitochondrial introns in both *osppr939-4* and *osppr939-5*. Primers across adjacent exons and across adjacent exons and introns were used to amplify the intron-spliced fragments and unspliced fragments, respectively. Splicing efficiency was calculated as the ratio of spliced to unspliced forms of each transcript in the mutants normalized to the same ratio in the wild type. The splicing efficiency of *nad5* introns 1, 2 and 3 was dramatically reduced in both mutants (Fig. 3d). Importantly, the significantly reduced splicing efficiency resulted from a reduction in *nad5* spliced forms (exon-exon) and an increase in *nad5* unspliced forms (exon–intron) (Fig. S9). However, other mitochondrial introns were spliced normally in *osppr939* (Fig. 3a, d). These results demonstrate that OsPPR939 participates in the *cis*-splicing of *nad5* intron 1 and the *trans*-splicing of *nad5* introns 2 and 3.

Although most PPR proteins involved in RNA editing belong to the PLS-subfamily, P-subfamily PPR proteins also play roles in RNA editing (Doniwa et al. 2010), suggesting that OsPPR939 might also function in the editing of mitochondrial transcripts. To investigate this possibility, we used RT-PCR and bulk sequencing of the amplified cDNAs to compare the editing efficiencies of mitochondrial transcripts in *osppr939-4* versus the wild type. Of the 491 mitochondrial RNA editing sites examined (Notsu et al. 2002), no significant difference in editing efficiency was observed between *osppr939-4* and the wild type. Hence, OsPPR939 is not essential for the editing of these RNAs in rice.

### Complementation of the *osppr939-4* mutant

**Fig. 4:**
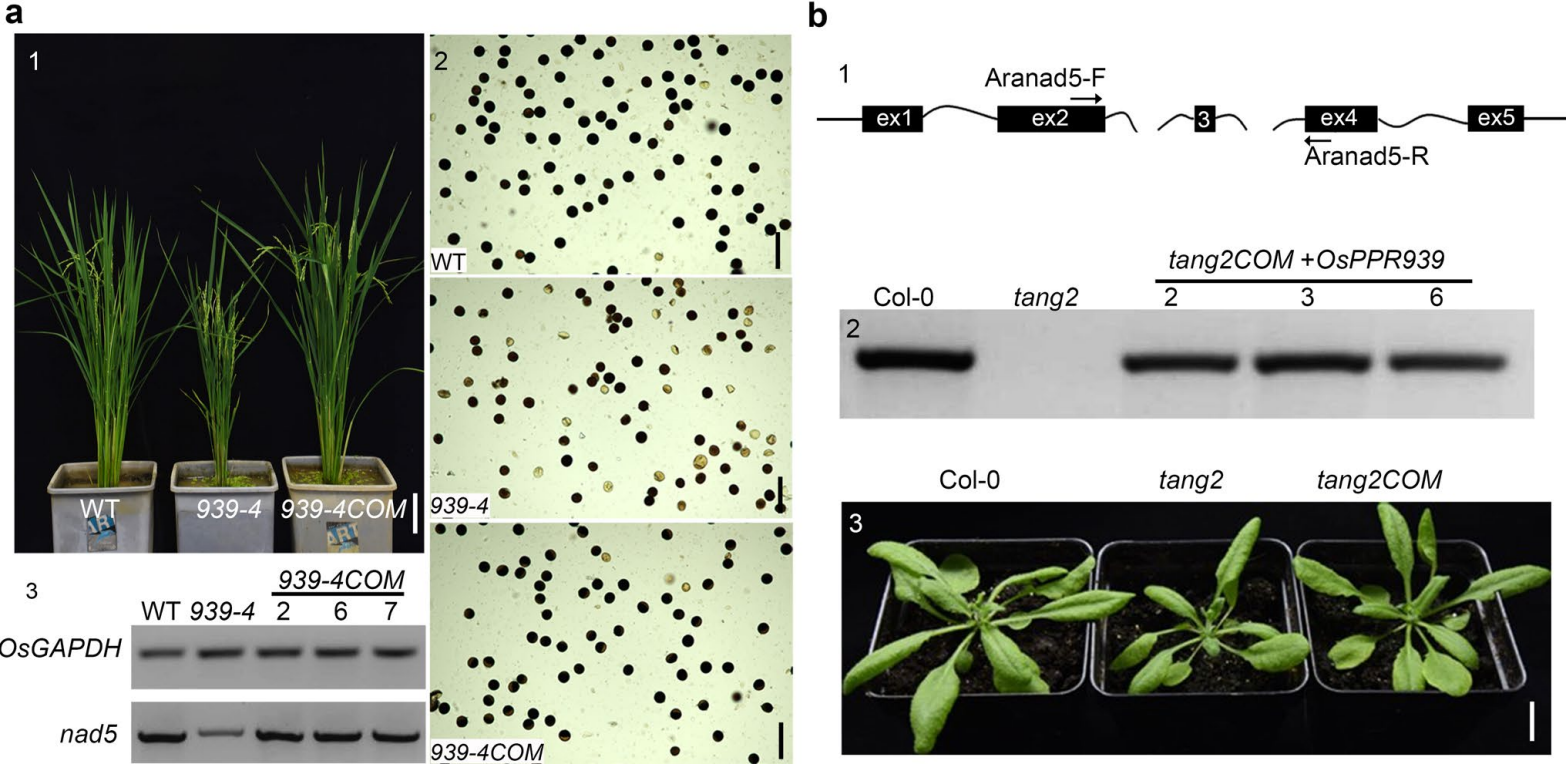
Complementation of *osppr939-4* and *tang2*. **a** Complementation with *OsPPR939pro:OsPPR93*9 rescued the defective phenotypes of *osppr939-4*. 1 Shows a wild type (WT) plant, *osppr939-4* plant, and *OsPPR939pro:OsPPR93*9-complemented (*osppr939-4COM*) plant expressing the wild type *OsPPR939* gene. 2 Shows I_2_-KI staining of pollen grains from wild type, *osppr939-4*, and *osppr939-4COM* plants. 3 Shows RT-PCR analysis of mature *nad5* transcripts from wild type, *osppr939-4*, and three complemented lines. *OsGAPDH* was used as a loading control. Bars = 10 cm (1), 100 μm (2). **b** *OsPPR939pro:OsPPR939* rescued the defective phenotypes of *tang2*. 1 Shows the structure of the *Arabidopsis* mitochondrial *nad5* gene. The positions of the primers used in 2 are shown. 2 Shows RT-PCR analysis of *Arabidopsis nad5* intron 3 spliced transcripts in Col-0, *tang2*, and three complemented lines expressing *OsPPR93*9. 3 Shows a Col-0 plant, *tang2* plant, and complemented *tang2*COM plant. Col-0, Columbia-0. Bars = 1 cm

To confirm that the mutant phenotypes and defective intron splicing were due to the lack of functional OsPPR939, we performed a complementation assay of *osppr939-4*. We amplified the native promoter along with the full-length coding sequence of *OsPPR939* from genomic DNA, inserted it into the pCAMBIA1300 vector, and introduced the construct into the *osppr939-4* mutant. In all seven independent positive transgenic lines, the *osppr939-4* phenotypes of reduced plant height and low pollen viability were successfully rescued (Fig. 4a, images 1 and 2, Fig. S8a, b). The reduced splicing efficiency of introns 1, 2, and 3 resulted in the weak accumulation of *nad5* transcript in the mutants (Fig. 3a, c). To investigate whether the splicing of these introns was recovered in the transgenic complementation lines, we performed RT-PCR to detect the mature *nad5* transcript using primer pair F1 and R4 (Fig. 3b). Complementation with *OsPPR939pro:OsPPR939* completely restored the intron splicing efficiency of the mutant to wild type levels (Fig. 4a, image 3). These results confirm that the defective intron splicing in the *osppr939* mutants is due to impaired OsPPR939 function, and that the abnormal growth is indeed caused by defects in intron splicing.

*Tang2*, the *Arabidopsis* ortholog of *OsPPR939*, is involved in the *trans*-splicing of *nad5* intron 3. Mutants of *Tang2* display retarded growth compared to the wild type. To investigate whether OsPPR939 could rescue the defective plant morphology of *tang2*, we transformed this mutant with the *OsPPR939pro:OsPPR939* construct for functional complementation. We performed RT-PCR using the primer pair Aranad5-F and Aranad5-R, located in exon 2 and 4, respectively (Fig. 4b, image 1), to examine the splicing efficiency of *nad5* intron 3. The results showed that intron 3-spliced transcripts were restored to wild type levels in the complemented lines (Fig. 4b, image 2), and the mutant phenotype of *tang2* was fully rescued (Fig. 4b, image 3). These data suggest that the functions of OsPPR939 and TANG2 are partially conserved.

### Mitochondrial complex I abundance and activity are reduced in *osppr939-4*

**Fig. 5:**
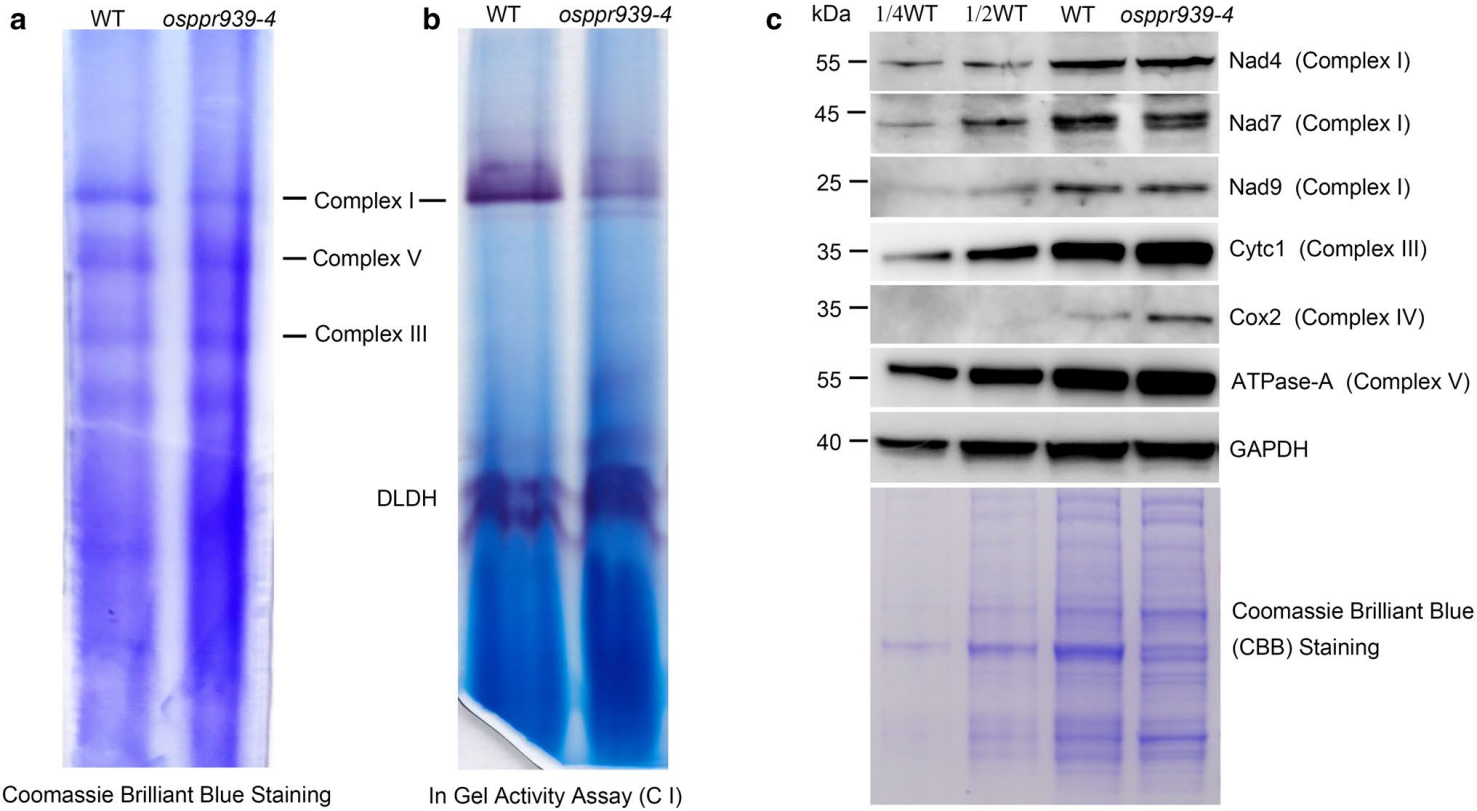
Complex I abundance and activity are reduced in the *osppr939-4* mutant. **a** BN gels stained with CBB. Mitochondrial complexes were isolated from rice calli and subjected to a 3% to 12% BN-PAGE. WT, wild type. **b** In-gel NADH dehydrogenase activity assay of mitochondrial complex I performed using dihydrolipoamide dehydrogenase*.* DLDH was used as a loading control. The positions of mitochondrial complexes are indicated. C I, complex I. **c** Total proteins extracted from wild type and *osppr939-4* calli were used for immunoblot analysis with antibodies against Nad4, Nad7, and Nad9 of complex I, Cyt*c*_*1*_ of complex III, Cox2 of complex IV, and ATPase-A of complex V. All blots were loaded in the same order. OsGAPDH and CBB staining are shown as a sample loading control. The molecular weights of the protein markers are indicated on the left

The substantially reduced *nad5* gene expression in *osppr939* mitochondria may result in the weak accumulation of Nad5 protein. Nad5 is a central subunit of mitochondrial complex I, one of the five complexes in the OXPHOS electron transport chain. To investigate the effect of the intron splicing deficiency of *nad5* on mitochondrial complex I, we examined complex I abundance by Blue Native Polyacrylamide Gel Electrophoresis (BN-PAGE) and complex I activity with an in-gel NADH dehydrogenase activity assay. We isolated crude mitochondria from *osppr939-4* and wild type calli and examined mitochondrial respiratory complex abundance by BN-PAGE. As indicated by Coomassie Brilliant Blue (CBB) staining, the abundance of complex I was reduced in *osppr939-4* compared to the wild type. The levels of both complex III and complex V were slightly higher in *osppr939-4* than in the wild type (Fig. 5a), perhaps due to responsible regulation, as described previously (Xiu et al. 2016). An in-gel NADH dehydrogenase activity assay confirmed that complex I activity was indeed dramatically reduced in *osppr939-4* (Fig. 5b).

We then measured the expression levels of several mitochondrial complex subunits by immunoblot analysis using total proteins extracted from calli. No difference was detected between wild type and *osppr939-4* proteins using antibodies against Nad4 and Nad9 (subunits of complex I), whereas the level of Nad7 (a subunit of complex I) was slightly reduced in the mutant. The levels of Cyt*c*_*1*_ (a subunit of complex III), Cox2 (a subunit of complex IV), and ATPase-α (a subunit of complex V) were higher in *osppr939-4* than in the wild type (Fig. 5c). Similar results were obtained using purified mitochondria for immunoblot analysis (Fig. S10).

**Fig. 6:**
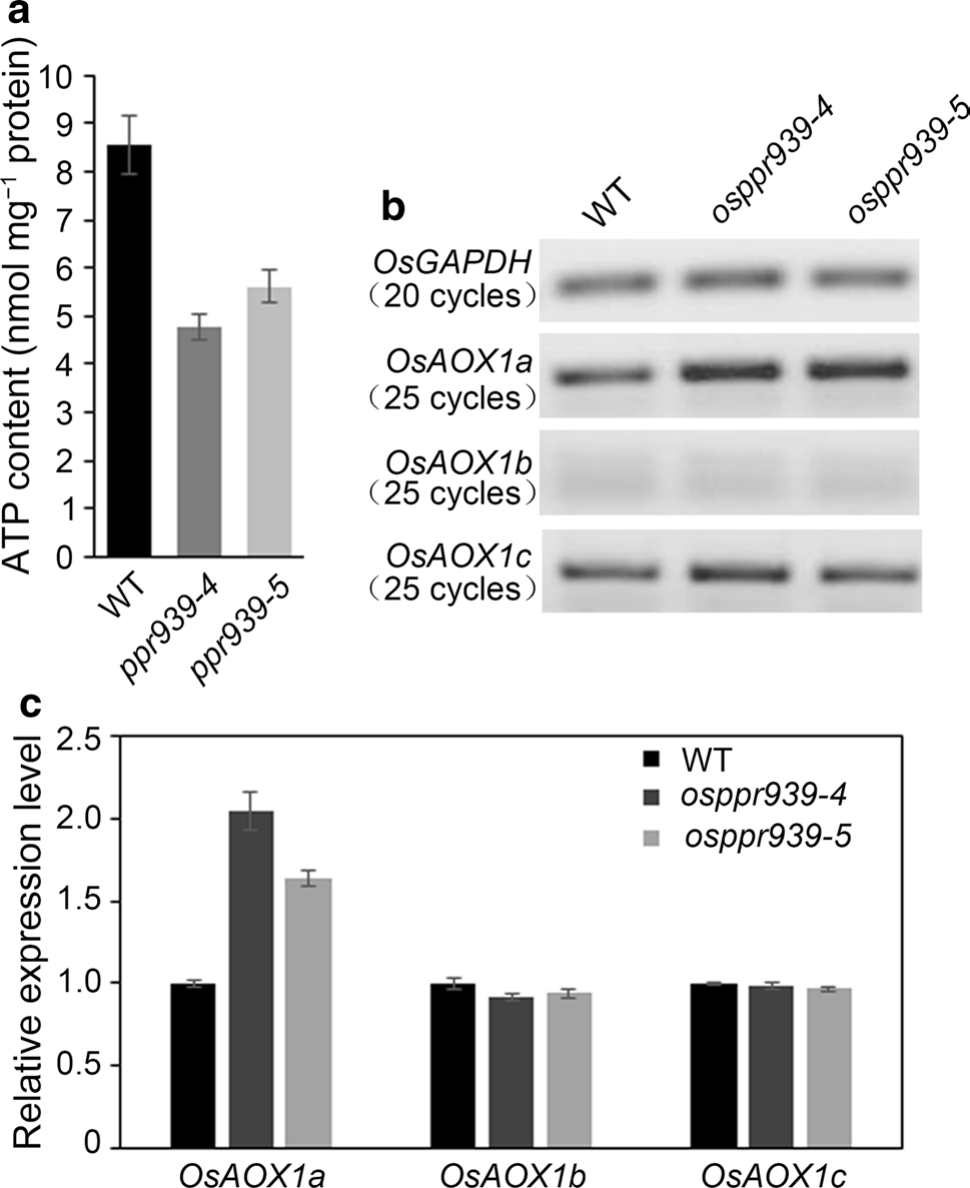
ATP contents and *OsAOX1a*, *OsAOX1b,* and *OsAOX1c* transcript levels in the *osppr939* mutants. **a** ATP contents of calli generated from mature seeds. Error bars indicate the SE based on three biological replicates. **b** RT-PCR analysis of *OsAOX1a* (AB004864.1), *OsAOX1b* (AB004865.1), and *OsAOX1c* (AB074005.1) transcripts in wild type (WT), *osppr939-4*, and *osppr939-5* plants. The numbers of PCR cycles are indicated on the left. The expression levels were normalized against *OsGAPDH*. **c** qRT-PCR analysis of *OsAOX1a*, *OsAOX1b*, and *OsAOX1c* transcripts. Error bars indicate SEs based on three biological replicates

Since ATP production in the mitochondria is coupled to electron transfer through complex I, we measured ATP levels in the calli. The ATP levels in *osppr939-4* and *osppr939-5* were only 55.9 and 65.6% that of the wild type, respectively (Fig. 6a). These results suggest that the lower level of mature *nad5* in the *osppr939* mutants due to the reduced splicing efficiency of *nad5* introns 1–3 indeed leads to reduced levels of complex I, which might stimulate the accumulation of other mitochondrial complexes via unknown retrograde signaling.

### Accumulation of alternative respiratory pathway transcripts in *osppr939*

A previously described splicing defect of *nad* transcripts led to partial or complete loss of complex I activity and thus increased levels of alternative oxidases (*AOX*) (de Longevialle et al. 2007; Xiu et al. 2016). To investigate whether the *nad5* splicing defect had similar effects in the *osppr939* mutants, we performed RT-PCR to measure the expression levels of *AOX* genes, including *AOX1a*, *AOX1b*, and *AOX1c*, in wild type and *osppr939* plants. The transcript levels of one of three AOX genes, *AOX1a,* were considerably higher in the mutants compared to the wild type (Fig. 6b). This result was further confirmed by qRT-PCR (Fig. 6c). We also performed qRT-PCR to measure the transcript levels of alternative NADH dehydrogenase genes (*NDA1-2*, *NDB1-3*, and *NDC1*). *NDA2* and *NDB3* transcript levels were higher in *osppr939-4* than the wild type (Fig. S11). Taken together, these results indicate that the loss of OsPPR939 function results in the induction of the alternative respiratory pathway by increasing the expression of related genes in the mutants.

#### OsS6K1 phosphorylates the N-terminal sequence of OsPPR939

**Fig. 7:**
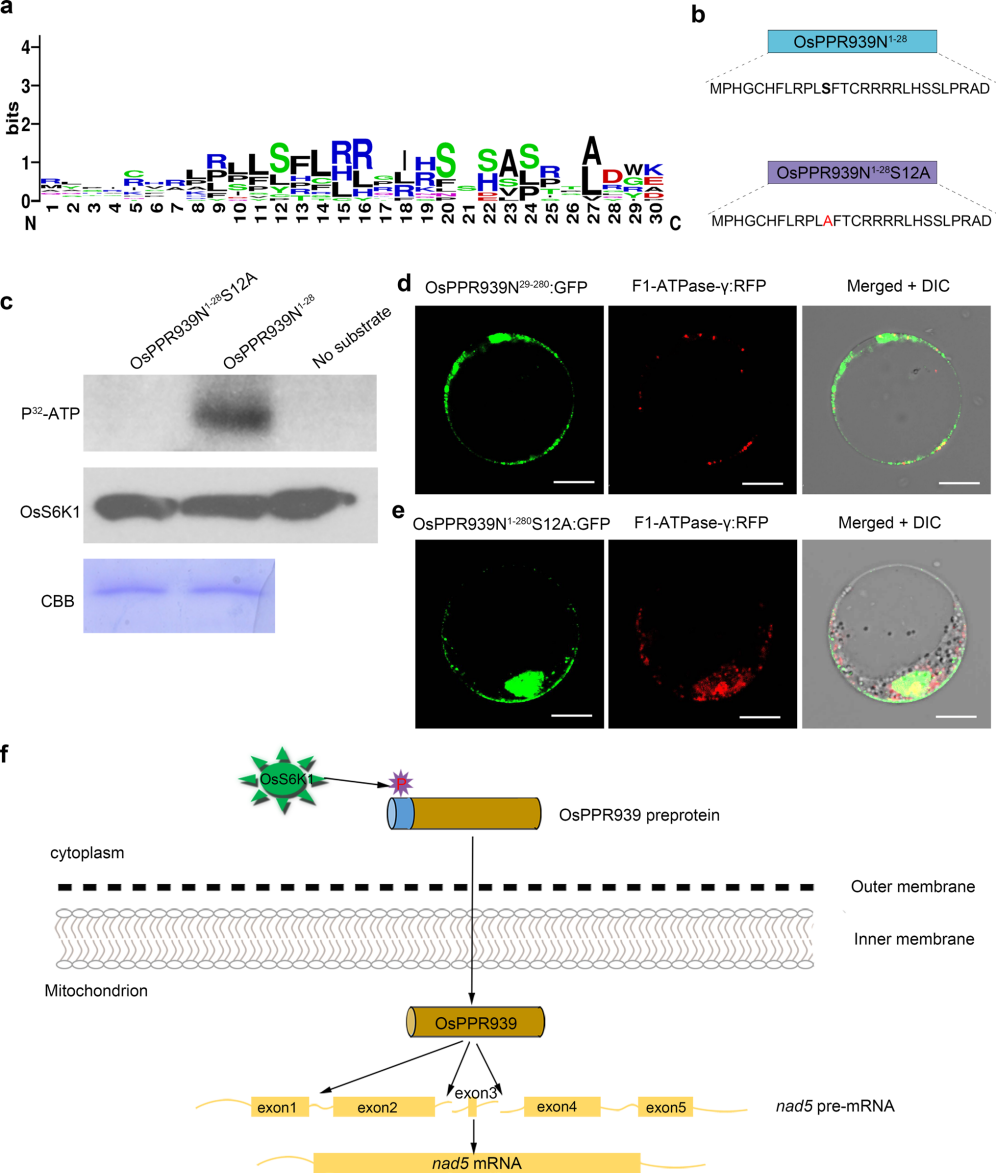
Mitochondrial import of OsPPR939 is regulated by phosphorylation. **a** Logo analysis of the 30 N-terminal amino acids of OsPPR939 and its counterparts from 15 homologs. The analysis was performed using the website of http://weblogo.berkeley.edu/logo.cgi. **b** OsPPR939N^1−28^ and OsPPR939N^1−28^S12A peptide sequences. **c** Phosphorylation analysis of the N-terminal targeting sequence and its mutated form in OsPPR939 by OsS6K1 kinase. OsS6K1 was purified from rice protoplasts expressing OsS6K1-HA fusion protein. GST-fused substrates were expressed in *E. coli*, followed by purification. CBB, Coomassie Brilliant Blue staining of substrates as a loading control. OsS6K1 incubated with no substrate was used as a negative control. **d** and **e** Subcellular localization analysis of OsPPR939N^29−280^:GFP (**d**) and OsPPR939N^1−280^S12A:GFP (**e**) by coexpression with F1-ATPase-γ:RFP in rice protoplasts. Bars = 5 μm. **f** A working model for the role of OsPPR939 in *nad5* intron splicing. OsS6K1 kinase phosphorylates the targeting peptide of OsPPR939 to facilitate its import into mitochondria, where it functions in the splicing of *nad5* introns 1, 2, and 3, thereby affecting the formation of mature *nad5* transcript

The phosphorylation of the N-terminal targeting peptide of nucleus-encoded mitochondrial proteins regulates the import of these proteins (Law et al. 2018). To examine whether the presequence of OsPPR939 contains a phosphorylation site, we used the web server PPSP (http://ppsp.biocuckoo.org/) to examine the first 28 amino acid residues of this protein. Three residues in this region were predicted to be phosphorylation sites: S12, S22, and S23. To determine whether these potential phosphorylation sites are conserved among the 15 homologous proteins of OsPPR939, we analyzed the N-terminal sequences of the homologs using the WebLogo program (Crooks et al. 2004) to create a sequence logo. As shown in Fig. 7a, the predicted phosphorylation site S12 was highly conserved among the homologous proteins in the plant species examined.

To experimentally validate the predicted phosphorylation sites, we expressed the N-terminal targeting peptide (OsPPR939N^1−28^) and the mutated peptide with the 12th residue changed from Ser-to-Ala (OsPPR939N^1−28^S12A) in *E. coli*, followed by purification (Fig. 7b). We then examined the abilities of several kinases to phosphorylate the S12 site. Among the kinases tested, only the kinase OsS6K1 phosphorylated the targeting peptide (Fig. S12), and OsS6K1 only phosphorylated the peptide OsPPR939N^1−28^, and not the mutated form (Fig. 7c).

To evaluate the importance of the first 28 residues and the phosphorylation of S12 for the mitochondrial localization of OsPPR939, we expressed two forms of GFP fusion protein, OsPPR939N^29−280^:GFP and OsPPR939N^1−280^S12A:GFP, in rice protoplasts with the mitochondrial reporter F1-ATPase-γ:RFP. As shown in Fig. 7d and e, most GFP signals did not overlap with RFP signals in the mitochondria, indicating that deleting the first 28 amino acid residues or the lack of phosphorylation at S12 seriously impeded the import of OsPPR939 into mitochondria.

To confirm that the impaired mitochondrial localization of OsPPR939N^1−280^S12A was not due to the Ser-to-Ala substitution, we examined the phosphorylation of the mutated peptides OsPPR939N^1−28^S12T and OsPPR939N^1−28^S12R, with the 12th residue Ser replaced by Thr or Arg, respectively (Fig. S12a). OsPPR939N^1−28^S12T but not OsPPR939N^1−28^S12R was phosphorylated by OsS6K1 (Fig. S13a). Finally, we detected the subcellular localization of the corresponding GFP fusion proteins in rice protoplasts. The GFP signals from both OsPPR939N^1−280^S12T:GFP and OsPPR939N^1−280^S12R:GFP colocalized with RFP signals from mitochondrial markers (Fig. S13C and D), indicating that the presence of a phosphorylated or positively charged residue at the N-terminus is essential for the mitochondrial localization of OsPPR939. Together, these results suggest that phosphorylation of the S12 in N-terminal targeting sequence of OsPPR939 is important for its mitochondrial localization.

## Discussion

Introns are rather common in the mitochondrial genomes of land plants, with 22 in the maize mitochondrial genome and 23 in *Arabidopsis* and rice (Bonen 2008). Because plant mitochondrial introns cannot undergo self-splicing, they require the help of nucleus-encoded splicing cofactors, which interact with intron RNA to catalyze the formation of its active conformation (Brown et al. 2014). So far, most PPR proteins acted as splicing factors in mitochondria have been identified in *Arabidopsis* and maize. In this study, we demonstrated that the P-subfamily PPR protein OsPPR939 is required for *cis*-splicing of *nad5* intron 1 and *trans-*splicing of *nad5* introns 2 and 3 in rice mitochondria. Two *osppr939* mutants (*osppr939-4* and *osppr939-5*) showed identical defects in the splicing of *nad5* introns, whereas other mitochondrial introns were correctly spliced in the mutants (Fig. 3), suggesting that a single *osppr939* mutation impaired the splicing of three *nad5* introns. By contrast, TANG2, the homolog of OsPPR939 in *Arabidopsis*, is specifically involved in *trans*-splicing of *nad5* intron 3 (des Francs-Small et al. 2014). Our findings indicate that the molecular functions of these two proteins are only partially conserved.

In *Arabidopsis,* the splicing of *nad5* intron 3 is impaired in *tang2* mutants, six-week-old mutant plants exhibit dark curled foliage when grown under a 16-h light/8-h dark photoperiod (des Francs-Small et al. 2014). In rice *osppr939* mutants, the splicing of *nad5* intron 1, 2, and 3 are defective, which results in far more serious phenotypes (Fig. 1b–h, Tables 1 and 2). The finding that mutations at different sites of a single gene cause mutant phenotypes of different severities has been widely documented (Cushing et al. 2005; Huang et al. 2018). One possible explanation is that mutations at different sites of a coding sequence impair gene function to different extents, with some mutations totally abolishing gene expression and others resulting in only a partial reduction in gene function, leading to very different morphological consequences. In this study, to explore the biological function of *OsPPR939* in “ideal” homozygous mutants (with severe but viable phenotypes), we targeted five sites for CRISPR/Cas9-mediated mutation. A mutation at the 5′-terminus (MS1) or middle regions (MS2 and MS3) of *OsPPR939* yielded no homozygous mutants, but homozygous mutants were obtained when a mutation occurred at the 3′-terminus (MS4 or MS5) of *OsPPR939* (Table 1). Genetic experiments demonstrated that pollen grains with the *osppr939* genotype from *osppr939-1* and *osppr939-2* heterozygous mutants were nonviable (Table 2). However, pollen grains with a mutated *OsPPR939* gene from *osppr939-3* could complete the process of fertilization, but the resulting zygotes failed to develop. By contrast, homozygous mutants *osppr939-4* and *osppr939-5* showed weaker compromised phenotypes, with only partially sterile pollen and reduced plant height (Fig. 1b–h, Fig. S2). These results imply that it is possible to obtain completely male sterile rice plants by knocking out *OsPPR939* to some extent.

Amino acids at specific positions in each PPR motif ensure that a given PPR protein can bind to single-stranded RNA in a sequence-specific fashion (Yin et al. 2013). Perhaps the severely defective phenotypes of the mutants could be explained by the following example. The *osppr939-1* mutant produces a truncated OsPPR939 protein with only three intact PPR motifs, which is not sufficient to allow the protein to recognize and splice the RNA sequence of *nad5.* As a result, no mature *nad5* RNA accumulates, leading to a complete loss of complex I activity, thereby resulting in pollen death. By contrast, *osppr939-4* and *osppr939-5* produce truncated OsPPR939 proteins with 16 and 18 motifs, respectively, which have retained partial functions for *nad5* intron splicing, resulting in reduced complex I abundance and partial pollen sterility. Overall, these findings indicate that *OsPPR939* plays crucial roles in plant growth and pollen development in rice, and that *osppr939-4* and *osppr939-5* are two weak alleles among the mutants created in this study.

Nad5 is one of nine Nad subunits of respiratory complex I (NADH:ubiquinone oxidoreductase) encoded by the mitochondrial genome (Meyer 2012). Before these *nad* mRNAs are translated into functional proteins, several post-transcriptional processes are required, including RNA editing, intron splicing, and RNA stability. Defects in any of these processes impair the assembly and activity of complex I. Several PPR proteins that participate in the intron splicing of *nad* mRNAs have been reported in *Arabidopsis* (de Longevialle et al. 2007; Liu et al. 2010; des Francs-Small et al. 2014; Hsieh et al. 2015; Haili et al. 2016) and maize (Xiu et al. 2016; Cai et al. 2017; Chen et al. 2017; Sun et al. 2018). The splicing defect of certain *nad* mRNAs leads to the partial or complete loss of complex I assembly and activity. In the current study, we determined that *OsPPR939* encodes a P-subfamily PPR protein that is required for the splicing of *nad5* introns 1, 2, and 3. The deficient splicing of these three introns in *osppr939* led to a complete or partial reduction in the levels of processed *nad5* transcript (Fig. 3a, c, d). As a result, mitochondrial complex I could not be assembled effectively due to lack or deficiency of the Nad5 subunit, and its activity was greatly weakened (Fig. 5a, b). This abnormality eventually resulted in growth retardation in the *osppr939* mutants, along with pollen death or partial sterility (Fig. 1b–f and Fig. S2, Tables 1 and 2). Likewise, the splicing defect of *nad5* intron 3 in *tang2* significantly reduces the amount of fully processed *nad5* transcript, resulting in severely reduced levels of complex I (des Francs-Small et al*.* 2014).

In mitochondria, complex I connects ATP synthesis with electron transfer (Millar et al. 2011). Defects in complex I block electron transfer, leading to a deficiency in oxidized NAD + , ultimately resulting in a loss in ATP production and changes in cellular metabolism. However, these abnormal signals can be also perceived by the alternative respiratory pathway, which is characterized by the expression of alternative NADH dehydrogenases and alternative oxidases (*AOX*) (Kühn et al*.* 2015). Several mutants with dysfunctional complex I that accumulate alternative NADH dehydrogenase and *AOX* transcripts have been reported (Keren et al. 2012; Xiu et al. 2016; Cai et al. 2017; Qi et al. 2017; Ren et al. 2017; Zhang et al. 2017; Sun et al. 2018; Sun et al. 2019). Consistent with these findings, we detected a significant reduction in ATP content in the *osppr939* mutants (Fig. 6a). Moreover, *AOX1a*, *NDA2*, and *NDB3* transcript levels were slightly higher in the *osppr939* mutants than the wild type (Fig. 6b, c and Fig. S10), indicating that the alternative respiratory pathway was activated in the mutants. Taken together, these data indicate that the defective phenotypes observed in *osppr939* plants were due to the reduced abundance of complex I.

Chloroplasts and mitochondria supply energy to living cells in plants. The biogenesis and functioning of these two organelles requires the involvement of many nucleus-encoded proteins. The N-terminal sequences of most nucleus-encoded preproteins determine their correct intracellular destinations (Chotewutmontri et al. 2012; Schleiff and Becker 2011). The phosphorylation of transit peptides of chloroplast proteins and the presequences of mitochondrial proteins functions in protein import between the cytosol and organelles (Law et al. 2015; Nickel et al. 2015). In the current study, we demonstrated that the 12th amino acid residue (Ser) on the N-terminal targeting peptide of OsPPR939 is phosphorylated by OsS6K1 (Fig. 7c), a member of the AGC family (PKA, PKG, and PKC) of Ser/Thr kinases (Jacinto and Lorberg 2008). The conserved kinase S6K1 is a major substrate and crucial effector of TOR (target of rapamycin) kinases (Wullschleger et al. 2006). In animals, “mTORC-S6K1” signaling controls fundamental cellular process by integrating multiple signals such as nutrient, stress, and energy signals (Magnuson et al. 2012). However, the regulatory mechanism of S6K1 in plants is poorly understood.

OsPPR939 is crucial for intron splicing of *nad5*, which encodes a core subunit of electron transport chain complex I. In mitochondria, a majority of ATP production is coupled to normal electron transfer. Therefore, the correct translocation of OsPPR939 to mitochondria is important for energy homeostasis. In the current study, we demonstrated that abolishing the phosphorylation of the 12th residue on the N-terminal sequence of OsPPR939 by inducing a Ser-to-Ala conversion largely impeded the localization of OsPPR939 to mitochondria (Fig. 7d). The deletion of the first 28 amino acids of OsPPR939 (Fig. 7e) also impaired the mitochondrial localization of OsPPR939, whereas the Ser-to-Thr-mutation did not (Fig. S13c). These results suggest that the phosphorylation of the N-terminal sequence of OsPPR939 is important for its mitochondrial localization (Fig. 7f).

Surprisingly, the truncated OsPPR939 protein with the 12th residue Ser mutated to a non-phosphorylatable Arg localized to the mitochondria (Fig. S13D), suggesting that the mitochondria import process of OsPPR939 is associated with a different mechanism. Indeed, the phosphorylation of transit peptides of some nucleus-encoded mitochondrial or chloroplast preproteins by specific kinases affects the efficiency of their import into intracellular destinations (Law et al. 2015; Lamberti et al. 2011a, b). In addition, mitochondrial targeting peptides are enriched in positively charged residues (Arg in particular). These residues are conducive to the formation of the positively charged face of a protein and provide net positive charges, which are important for the correct translocation of mitochondrial proteins (Emanuelsson and von Heijne 2001; Neupert and Herrmann 2007). Thus, perhaps the phosphorylation of the targeting peptide of OsPPR939 promotes its interaction with auxiliary factors to form a guidance complex for mitochondrial localization. Indeed, Arg residues provide preproteins with a more positive charge, which is important for the mitochondrial localization of many nucleus-encoded proteins. However, more precise assays, such as an in vitro mitochondria import assay, are needed to test this hypothesis.

Much is known about the role of PPR proteins in regulating the expression of organelle genes at the post-transcriptional level, but little is known about the post-translational regulation of PPR proteins. To our knowledge, the 12th residue Ser on OsPPR939 is the first experimentally proven phosphorylation site in PPR proteins. This finding provides not only a new insight into investigating how PPR proteins are regulated at post-translational level but also a theoretical basis for chemical emasculation and heterosis utilization. Torin-1 is a specific inhibitor of TOR that can modulate the activity of S6K1 (Ma and Blenis 2009; Thoreen et al. 2009; Schepetilnikov et al. 2011). Therefore, torin-1 can be used to inhibit the phosphorylation activity of TOR, which consequently impedes the phosphorylation of OsPPR939 by blocking the activity of OsS6K1 and makes OsPPR939 unable to correctly locate in mitochondria, thus leading to rice pollen sterility. In this way, we can easily obtain chemical induced sterile lines for hybrid production. In practical application, the advantage of chemical emasculation is that the preferred parental combination is not restricted by the relationship between male sterile line, restorer line and maintainer line, and male sterile line does not need to be selected by special program. But at present, this is only a theoretical assumption, and further research needs to be carried out on a large scale.

In conclusion, our results suggest that nucleus-encoded OsPPR939 controls the assembly of mitochondrial complex I and energy generation by participating in splicing of mitochondrial *nad5* introns 1, 2, and 3, thus playing an important regulatory role in rice plant growth and pollen development. In addition, the 12th amino acid in the N-terminal targeting sequence of OsPPR939 protein can be specifically phosphorylated by rice kinase OsS6K1, and phosphorylation of this site may plays an important role in the mitochondrial localization of OsPPR939. These results provide a theoretical basis for the creation of chemically induced male sterile line, and also point out a direction for investigation the regulatory mechanism of PPR proteins, especially those Rf-related PPRs, at the post-translational level.

## Supplementary Information

Below is the link to the electronic supplementary material.

Supplementary file1 (PDF 1686 kb)
