# Supplementary material for: OsPPR939, a nad5 splicing factor, is essential for plant growth and pollen development in rice
Source: Theor Appl Genet. 2021 Jan 2;134(3):923–40. doi: 10.1007/s00122-020-03742-6 (PMC7925476; doi:10.1007/s00122-020-03742-6)
Supplement: Supplementary file 1 — Supplementary file1 (PDF 1686 kb) [file 122_2020_3742_MOESM1_ESM.pdf]

OsPPR939, a *nad5* splicing factor, is essential for plant growth and pollen development in rice

Peng Zheng[1†], Yujun Liu[1†*], Xuejiao Liu[1], Yuqing Huang[1], Feng Sun[2], Wenyi Wang[1], Hao Chen[1], Mehmood Jan[1], Cuicui Zhang[3], Yue Yuan[1], Bao-Cai Tan[2], Hao Du[1*], Jumin Tu[1*]

1. Institute of Crop Science, Zhejiang University, Hangzhou 310058, China

2. Key Laboratory of Plant Development and Environmental Adaptation Biology, Ministry of Education, School of Life Sciences, Shandong University, Qingdao 266237, China

3. College of Life Science and Technology, Guangxi University, Nanning 530004, China

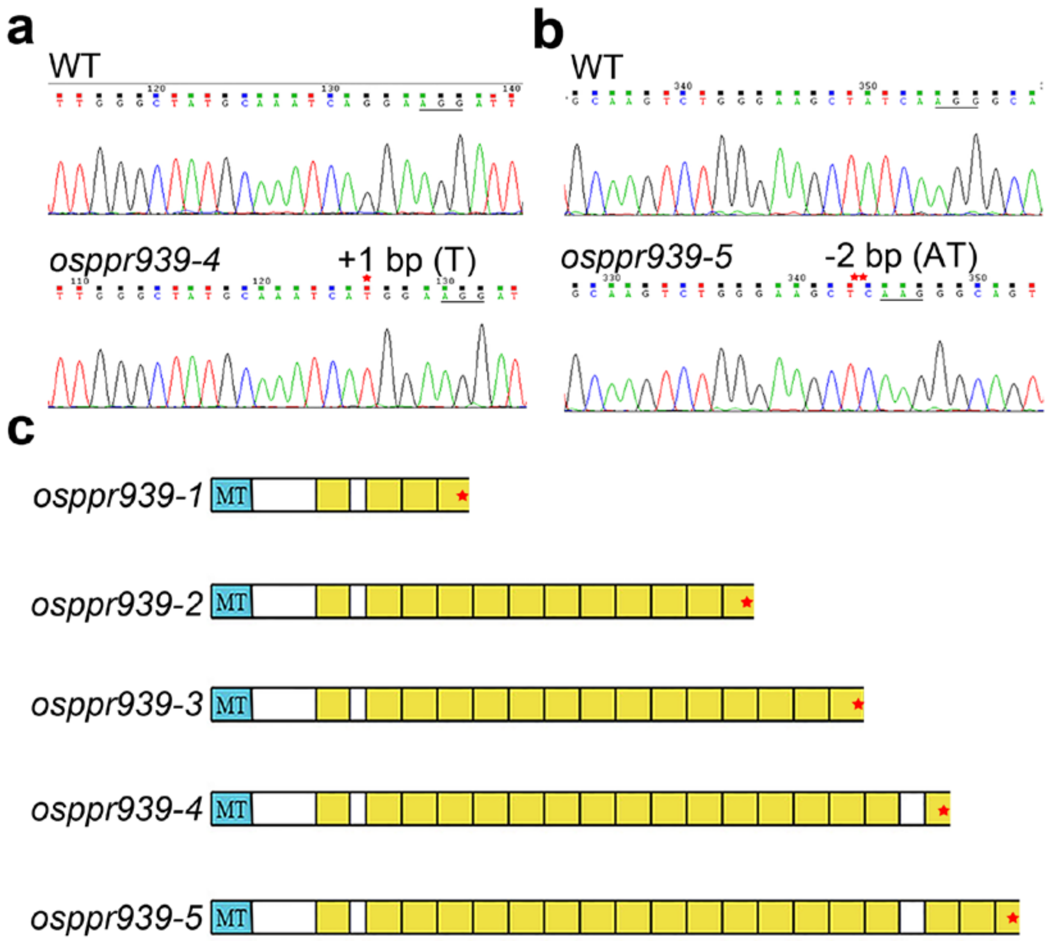

Fig. S1 Sequence confirmation and structures of the truncated proteins of the *osppr939* mutants **a** Sanger sequencing chromatograms of MS4 in a wild-type plant and in *osppr939-4*, which was produced by CRISPR/Cas9-mediated genome editing. The asterisks and underlined text indicate the inserted nucleotide and PAM sequences, respectively. **b** Sanger sequencing chromatograms of MS5 in a wild-type plant and in *osppr939-5*, which was produced by CRISPR/Cas9-mediated genome editing. The asterisks and underlined text indicate the deleted nucleotides and PAM sequences, respectively. **c** Schematic of truncated OsPPR939 proteins of the five mutants examined in this study. Asterisks indicate the termination site of each protein

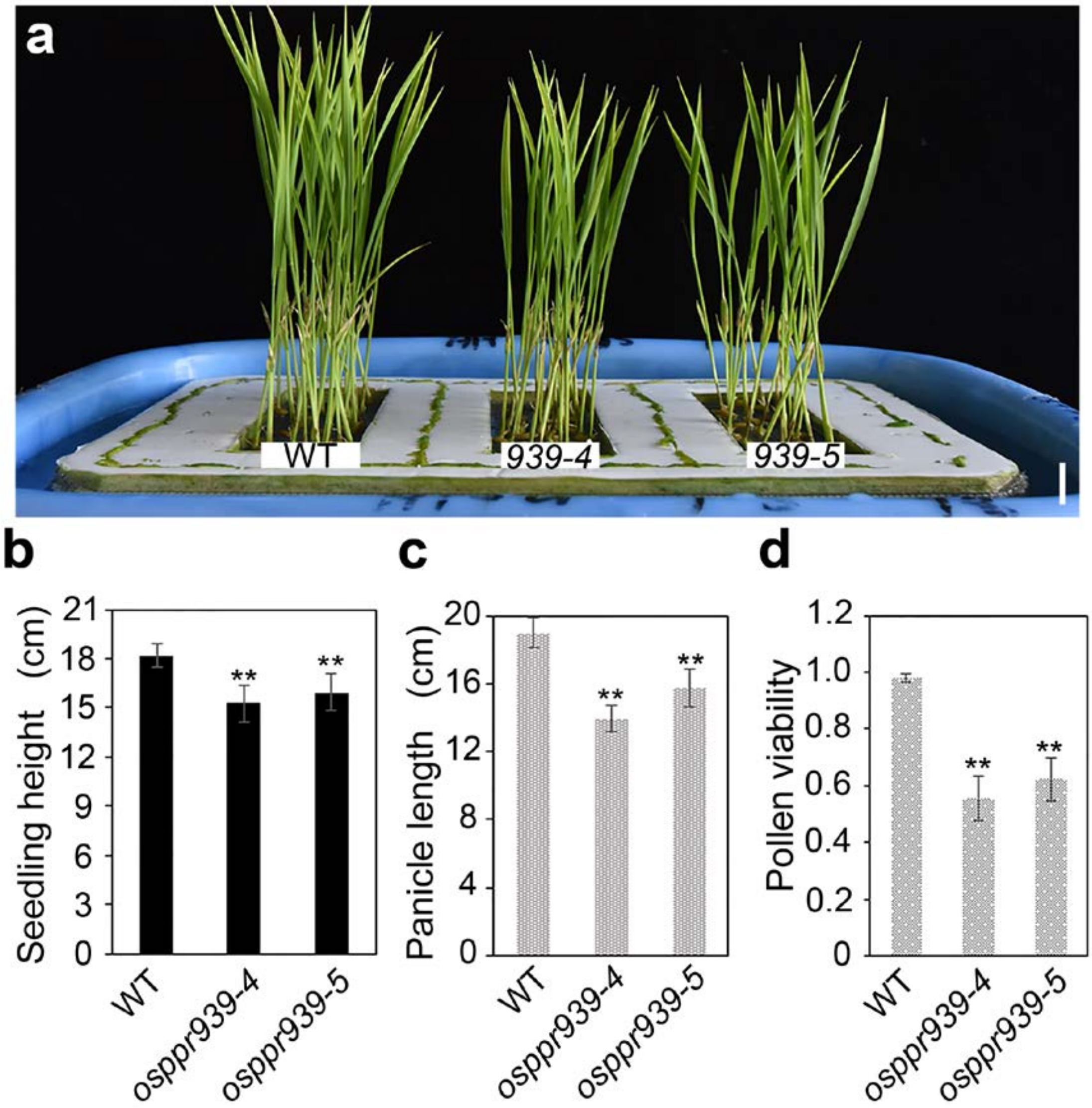

Fig. S2 Phenotypic characterization of *osppr939* seedlings. **a** Comparison of plant height in wild type (WT), *osppr939-4*, and *osppr939-5* plants at the seedling stage. (**b-d**) Comparison of various traits in WT and two *osppr939* mutants, including average plant height (n = 10 plants) (**b**), average panicle length (n = 20 plants) (**c**), and average pollen viability (n = 7 views) (**d**). Error bars indicate the SD. **, P< 0.01, by Student's t-test

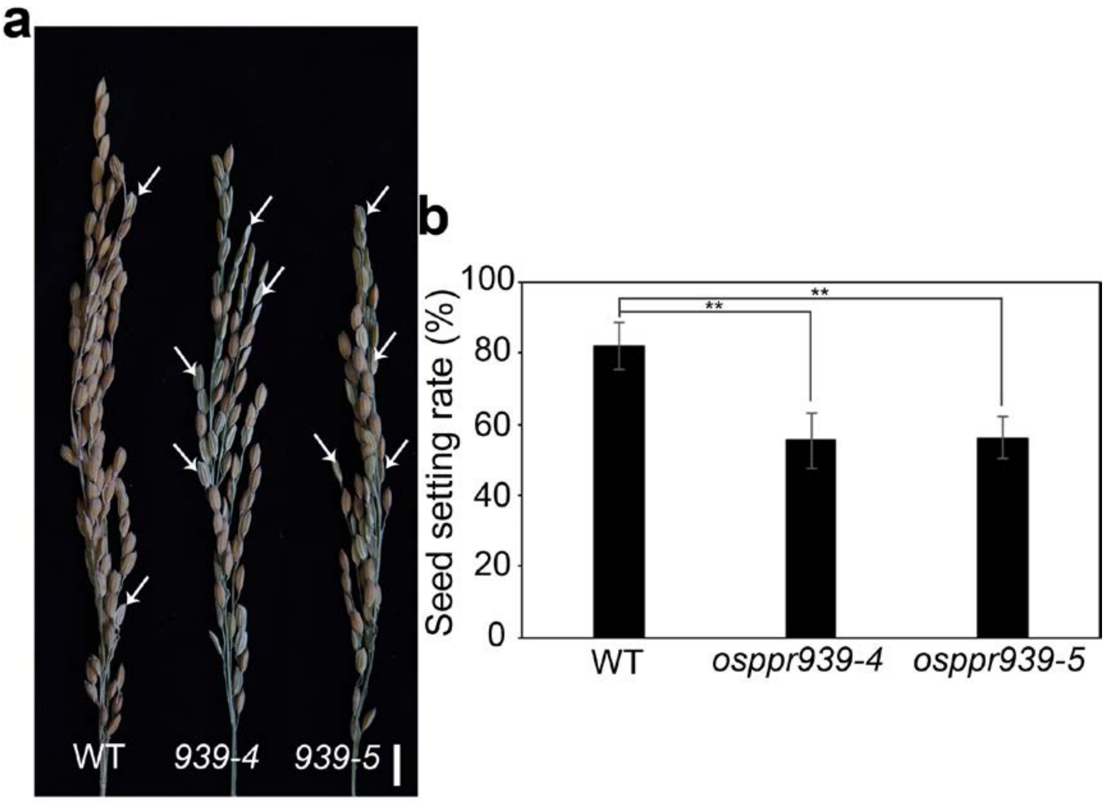

Fig. S3 Seed setting rates are reduced in the *osppr939* mutants. **a** Panicles of wild type and two *osppr939* mutants at the mature stage. Unfilled grains are indicated by white arrows. WT, wild type. Bars = 1 cm. **b** Seed setting rates of wild type and *osppr939* plants. Error bars indicate the SE based on 15 individual plants. **, $P< 0.01$, by Student's *t*-test

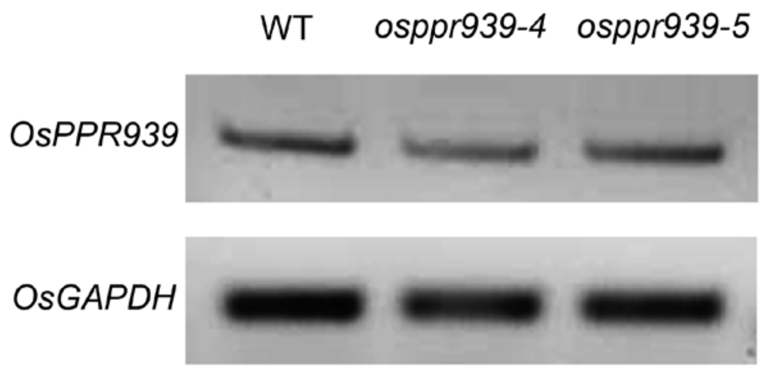

Fig. S4 Expression analysis of *OsPPR939* in the *osppr939* mutants. RT-PCR was used to detect the abundance of *OsPPR939* transcripts. *OsGAPDH* was used as a loading control. WT, wild type

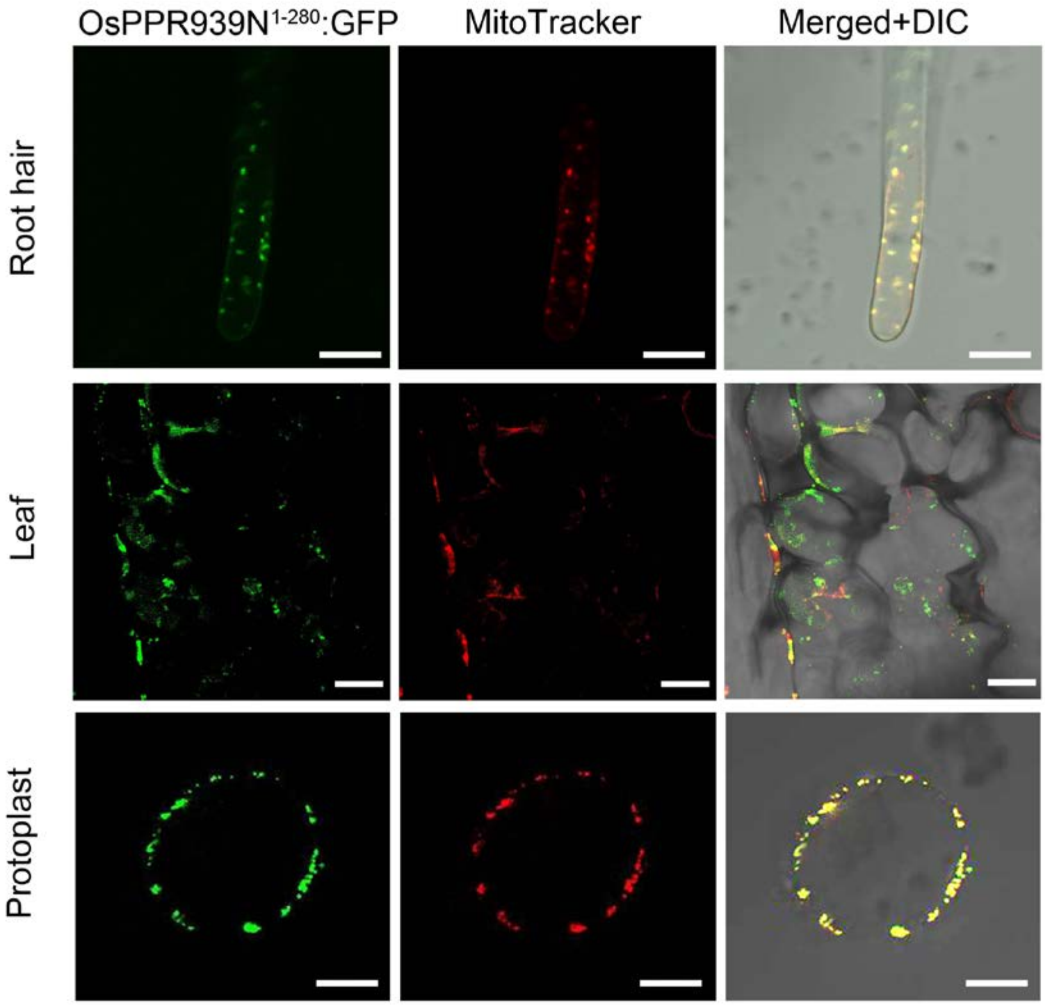

Fig. S5 Subcellular localization of OsPPR939 in *Arabidopsis*. Root hairs, leaves, and protoplasts from transgenic *Arabidopsis* plants stably expressing OsPPR939N$^{1-280}$:GFP fusion protein were stained with MitoTracker to label the mitochondria. Green and red fluorescent signals were observed by confocal microscopy. Root hairs and leaves were obtained from 1-week-old plants, while protoplasts were extracted from 4-week-old plants. DIC, differential interference contrast. Bars represent 10 µm (root hairs), 20 µm (leaves), 5 µm (protoplasts)

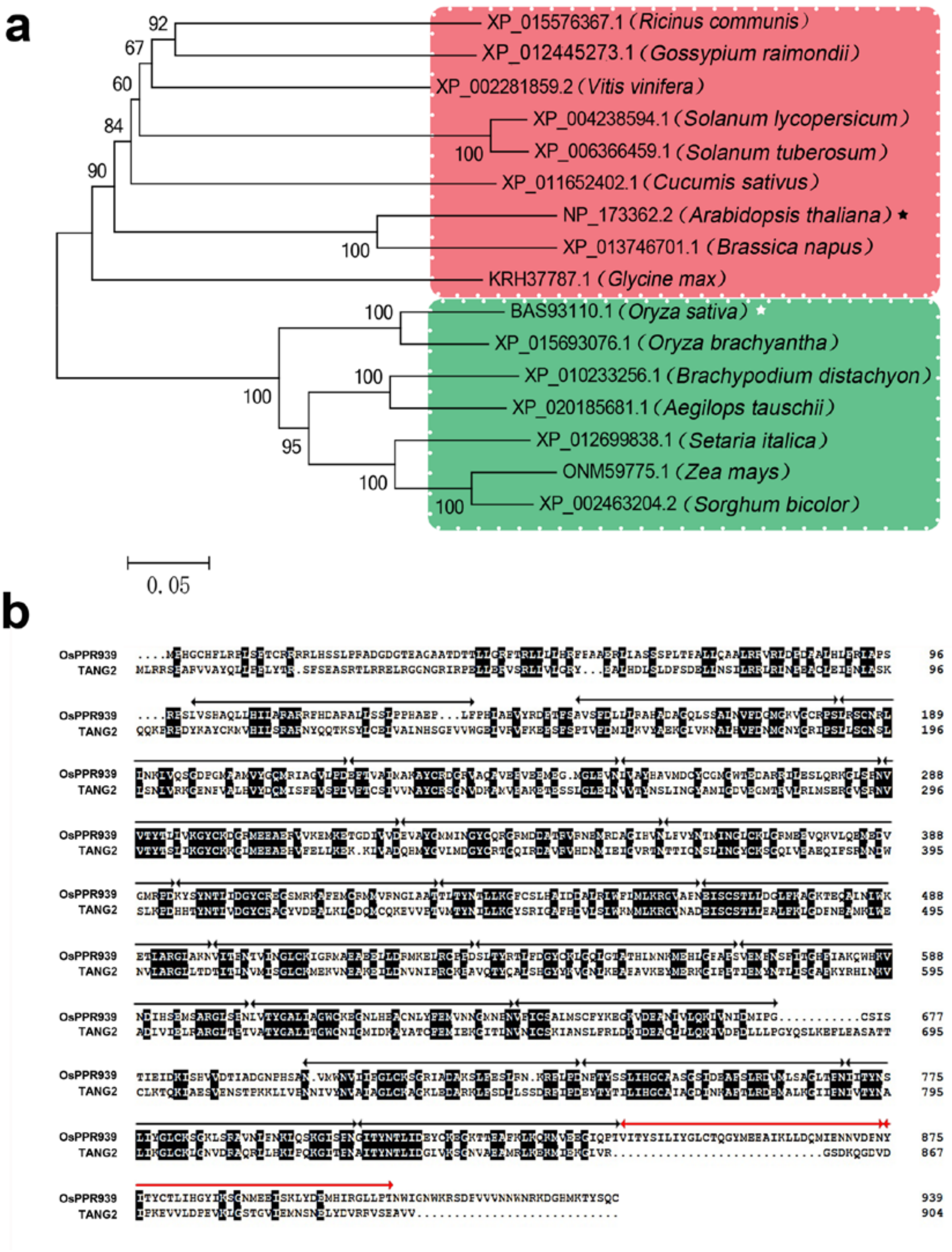

Fig. S6 Phylogenetic analysis of OsPPR939 homologs. **a** The phylogenetic analysis was performed by the neighbor-joining method using MEGA6.0 (Tamura et al., 2013). The numbers at the nodes represent the percentage of 1,000 bootstraps. The red box represents the dicotyledon cluster and the green box represents for monocotyledon cluster. The white and black stars indicate the positions of OsPPR939 and its homolog Tang2 in *Arabidopsis* in the tree, respectively. **b** Alignment of OsPPR939 and Tang2. The full-length amino acid sequences of both proteins were aligned with ClustalX2.0 (Larkin et al., 2007). Identical amino acids are shaded in black. PPR motifs that are conserved in the two proteins are indicated by black double-headed arrows, and those that are only present in OsPPR939 are indicated by red double-headed arrows

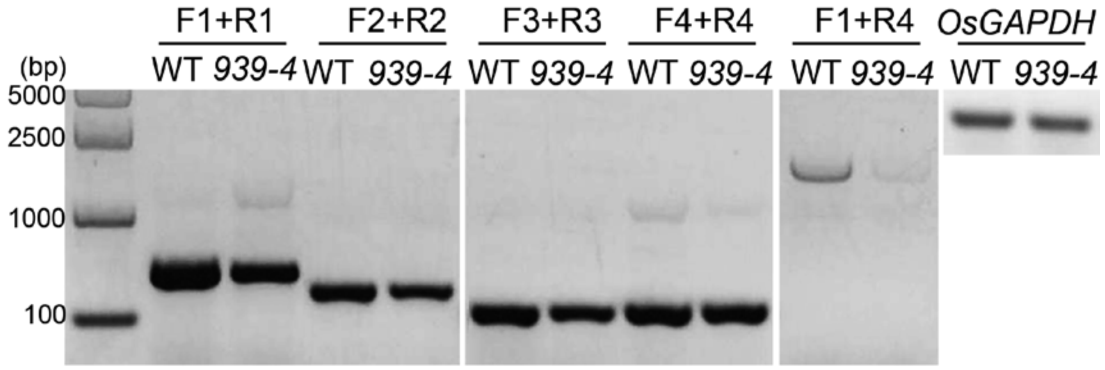

Fig. S7 RT-PCR analysis of *nad5* intron splicing in *osppr939-4* calli. RNA was isolated from wild type (WT) and *osppr939-4* calli. *OsGAPDH* was used as a loading control

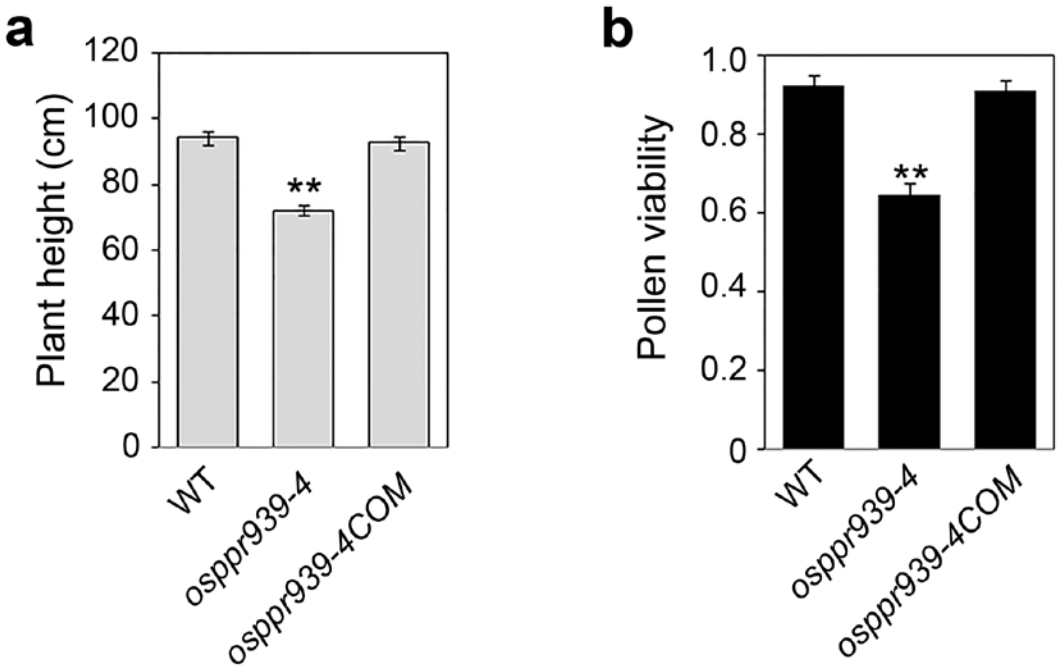

Fig. S8 The mutant phenotypes are restored in the complementation line. Comparisons between WT, *osppr939-4*, and complementation plants, including average plant height (n = 15 plants) (A) and average pollen viability (n = 8 views) (B). Error bars indicate the SD. **, P< 0.01, by Student's t-test

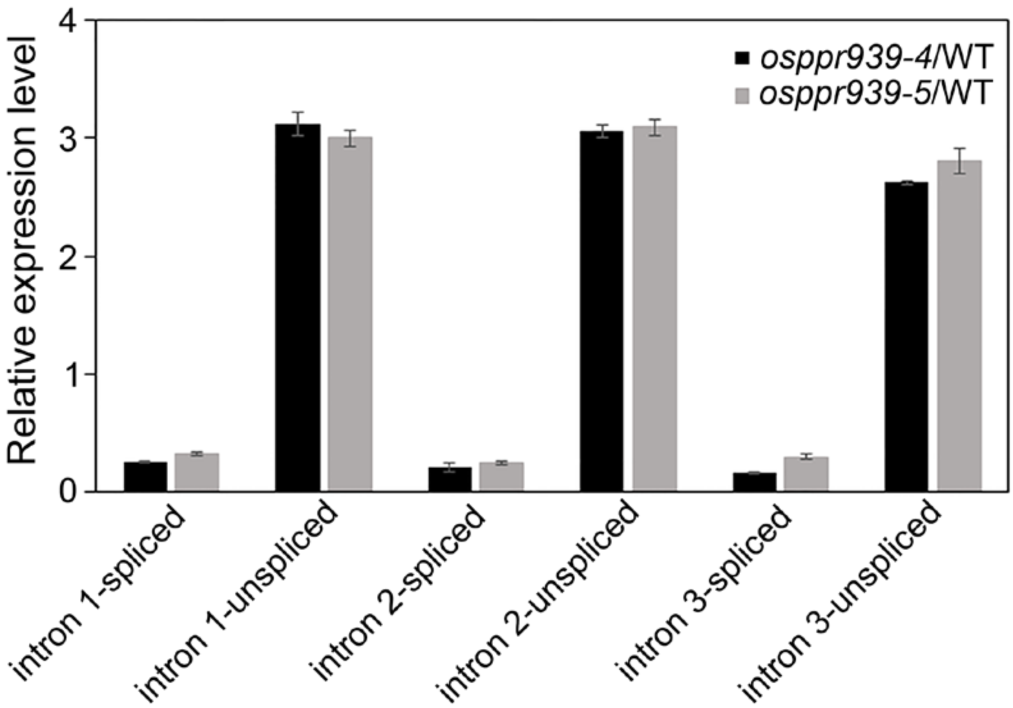

Fig. S9 qRT-PCR analysis of *nad5* transcript levels with or without introns 1, 2, or 3 in the *osppr939* mutants. The histogram shows the relative fold-change of *nad5* transcripts with (unspliced) or without (spliced) introns 1, 2, or 3 in *osppr939-4* and *osppr939-5* compared to the wild type (WT). The primers used for qRT-PCR analysis are listed in Table S2

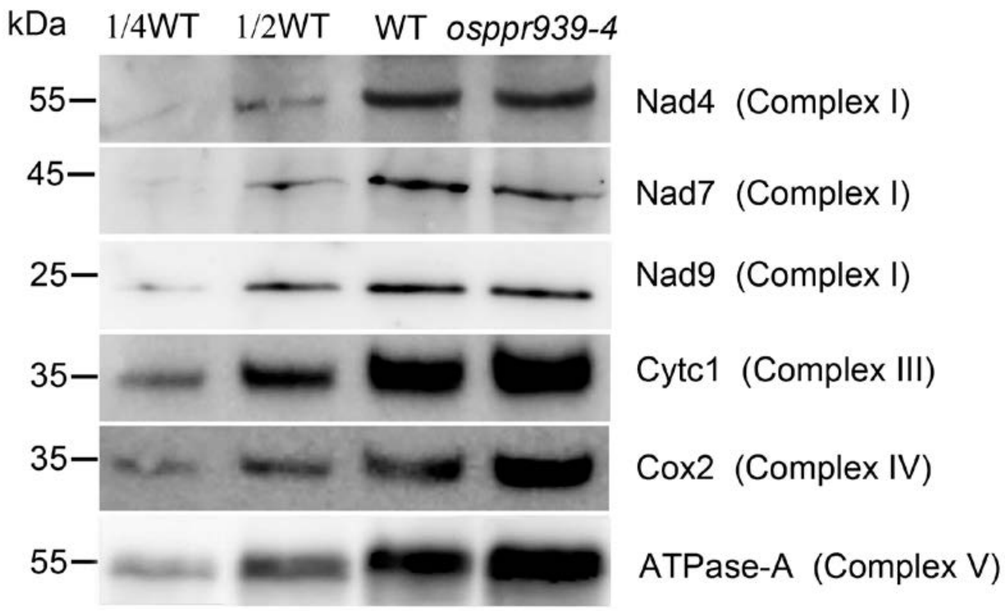

Fig. S10 The abundance of proteins in the mitochondrial respiration chain is affected in the *osppr939-4* mutant. Mitochondrial proteins (12 μg) extracted from wild-type and *osppr939-4* calli were subjected to SDS-PAGE, and the proteins were transferred to a PVDF membrane and probed with antibodies against Nad4, Nad7, and Nad9 of complex I, Cytc_1 of complex III, Cox2 of complex IV, and ATPase-A of complex V. All immunoblots were loaded in the same order. The molecular weights of the protein markers are indicated on the left

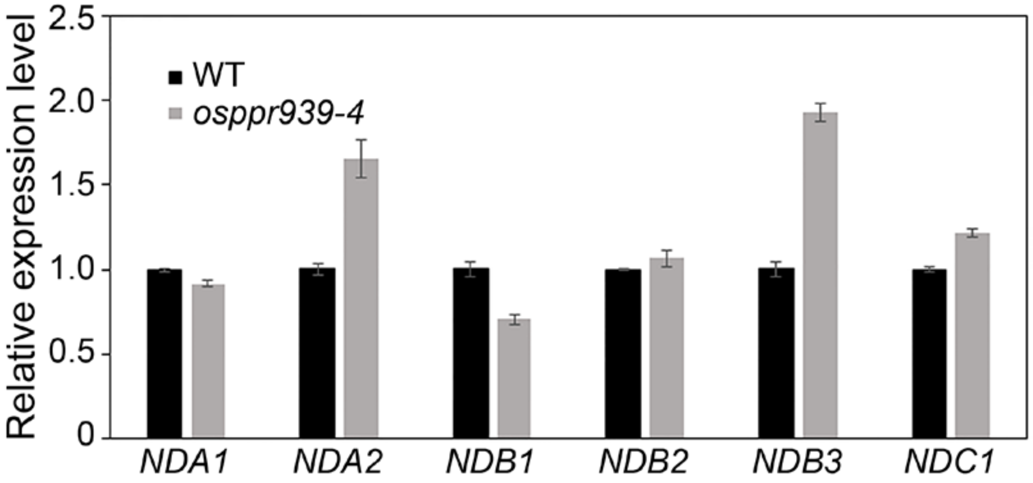

Fig. S11 Abundance of alternative NADH dehydrogenase-related transcripts in the *osppr939-4* mutant. qRT-PCR analysis of alternative external NADH dehydrogenase genes *NDB1* (LOC4341876), *NDB2* (LOC9266372), and *NDB3* (LOC4338442) and internal NADH dehydrogenase genes *NDA1* (LOC4327426), *NDA2* (LOC4343616), and *NDC1* (LOC4340478) in *osppr939-4* compared to the wild type (WT). Error bars indicate the SE based on three biological replicates

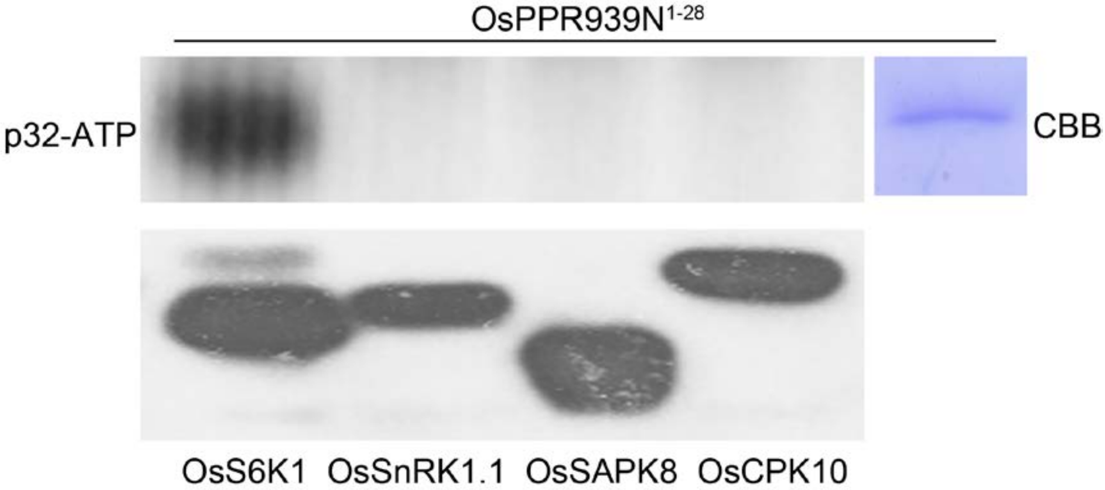

Fig. S12 Validation of candidate kinases of OsPPR939N$^{1\text{-}28}$ using in vitro kinase assays. OsS6K1, OsSnRK1.1, OsSAPK8, and OsCPK10 kinases were purified from rice protoplasts expressing HA-fused protein kinases. OsPPR939N$^{1\text{-}28}$ was fused to the GST tag and produced from *E. coli.* CBB, Coomassie brilliant blue staining of substrate as a loading control

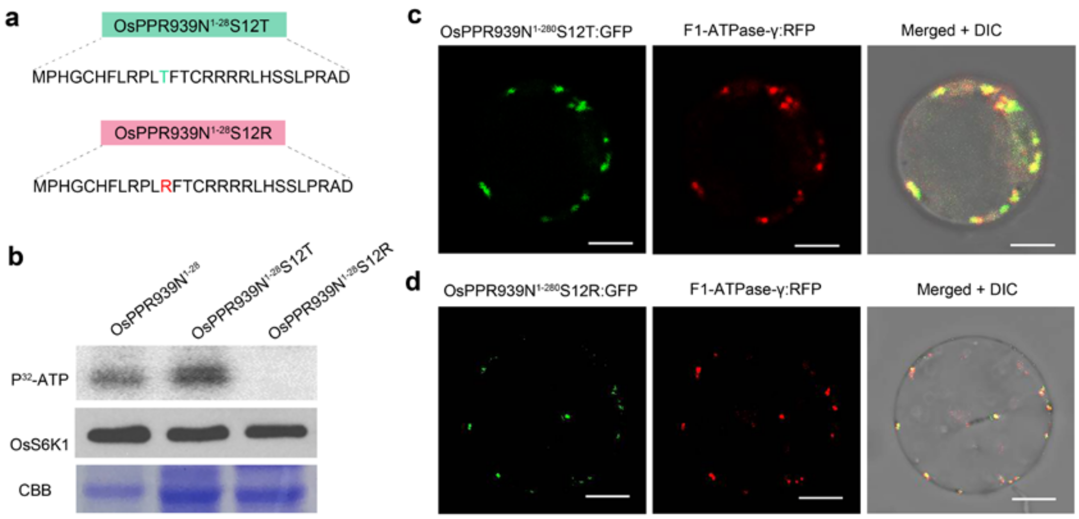

Fig. S13 Phosphorylation of OsPPR939N$^{1\text{-}28}$S12T and OsPPR939N$^{1\text{-}28}$S12R and subcellular localization of their corresponding GFP fusion proteins. **a** Peptide sequences of OsPPR939N$^{1\text{-}28}$S12T and OsPPR939N$^{1\text{-}28}$S12R. **b** Phosphorylation analysis of two mutated forms of the N-terminal targeting sequence of OsPPR939 by OsS6K1 kinase. OsS6K1 was purified from rice protoplasts expressing OsS6K1-HA fusion protein. GST-fused substrates were expressed in *E. coli*, followed by purification. CBB, Coomassie brilliant blue staining of substrates as a loading control. OsPPR939N$^{1\text{-}28}$ was used as a negative control. (**c, d**) Subcellular localization of OsPPR939N$^{1\text{-}280}$S12T:GFP (**c**) or OsPPR939N$^{1\text{-}280}$S12R:GFP (**e**) coexpressed with F1-ATPase-γ:RFP in rice protoplasts. Bars = 5 μm

Table S1. *osppr939* mutant lines created by CRISPR/Cas9.

**Reference**	AGCTAGTGCAGTCTGGGGATCCAGGCA-TGGCGGCCATGGTGTA	
**#MS1-24**		
Allele 1	AGCTAGTGCAGTCTG----------------CGGCCATGGTGTA	-15bp
Allele 2	AGCTAGTGCAGTCTGGGGATCCAGGCA----CGGCCATGGTGTA	-3bp
**#MS1-25 (*osppr939-1*)**		
Allele 1	AGCTAGTGCAGTCTGGGGATCCAGGC--TGGCGGCCATGGTGTA	-1bp
Allele 2	AGCTAGTGCAGTCTGGGGATCCAGGCA-TGGCGGCCATGGTGTA	WT

**Reference**	AATTGAGATGCCCTCCGG-ATAGCCTAACTTACAGAACATTATT	
**#MS2-1/10**		
Allele 1	AATTGAGATGCCCTCC------GCCTAACTTACAGAACATTATT	-5bp
Allele 2	AATTGAGATGCCCTCC----AGGCCTAACTTACAGAACATTATT	-3bp
**#MS2-2**		
Allele 1	AATTGAGATGCCCTCC-------CCTAACTTACAGAACATTATT	-6bp
Allele 2	AATTGAGATGCCCTCC------GCCTAACTTACAGAACATTATT	-5bp
**#MS2-5 (*osppr939-2*)**		
Allele 1	AATTGAGATGCCCTCCG--ATAGCCTAACTTACAGAACATTATT	-1bp
Allele 2	AATTGAGATGCCCTCCGG-ATAGCCTAACTTACAGAACATTATT	WT
**#MS2-14**		
Allele 1	AATTGAGATGCCCTCCGGGATAGCCTAACTTACAGAACATTATT	+1bp
Allele 2	AATTGAGATGCCCTCCAA-GAGGGGGATGGATAAAGTGGGCAAA	+156bp

**Reference**	TAATTTAGTCACTTATGGAGCTCTGATAGCTGGATGGTGCAAA	
**#MS3-5**		
Allele 1	TAATTTAGTCACTTATGGAGCT-----AGCTGGATGGTGCAAA	-5bp
Allele 2	TAATTTAGTCACTTATGGAGC------AGCTGGATGGTGCAAA	-6bp
**#MS3-6/7**		
Allele 1	TAATTTAGTCACTTATGGAGCT-----AGCTGGATGGTGCAAA	-5bp
Allele 2	TAATTTAGTCACTTATGGAGCTCT---AGCTGGATGGTGCAAA	-3bp
**#MS3-8/14**		
Allele 1	TAATTTAGTCACTTATGGAGCTCT---AGCTGGATGGTGCAAA	-3bp
Allele 2	TAATTTAGTCACTTATGGAGCTCT---AGCTGGATGGTGCAAA	-3bp
**#MS3-9**		
Allele 1	TAATTTAGTCACTTATGGAGC-------------------AAA	-19bp
Allele 2	TAATTTAGTCACTTATGGAGCTCT---AGCTGGATGGTGCAAA	-3bp
**#MS3-11**		
Allele 1	TAATTTAGTCACTTATGGAGCTC-----GCTGGATGGTGCAAA	-5bp
Allele 2	TAATTTAGTCACTTATGGAGCT---ATAGCTGGATGGTGCAAA	-3bp
**#MS3-13**		
Allele 1	TAATTTAGTCACTTATGG-------------------TGCAAA	-19bp
Allele 2	TAATTTAGTCACTTATGGAGCTCT---AGCTGGATGGTGCAAA	-3bp
**#MS3-16 (*osppr939-3*)**		

Allele 1	TAATTTAGTCACTTATGGAGCTCTGA-AGCTGGATGGTGCAAA	-1bp
Allele 2	TAATTTAGTCACTTATGGAGCTCTGATAGCTGGATGGTGCAAA	WT
**Reference**	GTTATTATATTTGGGCTATGCAAATCA-GGAAGGATTGCAGATG	
**#MS4-3 (*osppr939-4*)**		
Allele 1	GTTATTATATTTGGGCTATGCAAATCATGGAAGGATTGCAGATG	+1bp
Allele 2	GTTATTATATTTGGGCTATGCAAATCATGGAAGGATTGCAGATG	+1bp
**#MS4-7/8**		
Allele 1	GTTATTATATTTGGGCTATGCA----------GGATTGCAGATG	-9bp
Allele 2	GTTATTATATTTGGGCTATGCAAA--------GGATTGCAGATG	-7bp
**#MS4-13**		
Allele 1	GTTATTATATTTGGGCTATGCA---------AGGATTGCAGATG	-8bp
Allele 2	GTTATTATATTTGGGCTATGCAAATCAAGGAAGGATTGCAGATG	+1bp
**#MS4-15**		
Allele 1	GTTATTATATTTGGGCTATGCAAATC--GGAAGGATTGCAGATG	-1bp
Allele 2	GTTATTATATTTGGGCTATGCAAATC------------------	-17bp
**#MS4-20**		
Allele 1	GTTATTATATTTGGGCTATGCAAATCAAGGAAGGATTGCAGATG	+1bp
Allele 2	GTTATTATATTTGGGCTATGCAAATCAAGGAAGGATTGCAGATG	+1bp
**#MS4-21/22**		
Allele 1	GTTATTATATTTGGGCTATGCAAATCAAGGAAGGATTGCAGATG	+1bp
Allele 2	GTTATTATATTTGGGCTATGCAAATCATGGAAGGATTGCAGATG	+1bp
**#MS4-23**		
Allele 1	GTTATTATAT-----CT------------------------ATG -30bp/+2bp	
Allele 2	GTTATTATATTTGGGCTATGCAAA----GGAAGGATTGCAGATG	-3bp
**Reference**	TATGGCCTCTGCAAGTCTGGGAAGCT-ATCAAGGGCAGTTAACC	
**#MS5-5**		
Allele 1	TATGGCCTCTGCAAGTCTGGGA------TCAAGGGCAGTTAACC	-5bp
Allele 2	TATGGCCTCTGCAAGTCTGGGAAGC---TCAAGGGCAGTTAACC	-2bp
**#MS5-9/12**		
Allele 1	TATGGCCTCTGCAAGTCTGGGAA-----TCAAGGGCAGTTAACC	-4bp
Allele 2	TATGGCCTCTGCAAGTCTGGGAAGCT--TCAAGGGCAGTTAACC	-1bp
**#MS5-11/17**		
Allele 1	TATGGCCTCTGCAAGTCTGGGAA-----TCAAGGGCAGTTAACC	-4bp
Allele 2	TATGGCCTCTGCAAGTCTGGGAAGC---TCAAGGGCAGTTAACC	-2bp
**#MS5-14 (*osppr939-5*)**		
Allele 1	TATGGCCTCTGCAAGTCTGGGAAGC---TCAAGGGCAGTTAACC	-2bp
Allele 2	TATGGCCTCTGCAAGTCTGGGAAGC---TCAAGGGCAGTTAACC	-2bp
**#MS5-18/21/23/24/26**		
Allele 1	TATGGCCTCTGCAAGTCTGGGAAGC---TCAAGGGCAGTTAACC	-2bp
Allele 2	TATGGCCTCTGCAAGTCTGGGAAGCT--TCAAGGGCAGTTAACC	-1bp
#MS5-25		

Allele 1	TATGGCCTCTGCAAGTCTGGGAAGCT------GGGCAGTTAACC	−5bp
Allele 2	TATGGCCTCTGCAAGTCTGGGAAGCT---CAAGGGCAGTTAACC	−2bp
**#MS5-28**		
Allele 1	TATGGCCTCTGCAAGTCTGGGAAGCT-A-CAAGGGCAGTTAACC	−1bp
Allele 2	TATGGCCTCTGCAAGTCTGGGAAGCTTATCAAGGGCAGTTAACC	+1bp

The reference sequence is shown at the top, with the PAM sequence shown in blue and the target sequence shown in red. Black dashes indicate deleted bases, and the five *osppr939* mutants used in this study are shown in green. The net change in length is indicated to the right of each sequence (+, insertion; –, deletion). Note that some alleles have both sequence insertions and deletions.

Table S2. Primers used in this study.

**Prime name**	**Primer sequence (5′→3′)**	**use**
GT634-F	CGCCGAGGTCTACAGGGACT	Identify *osppr939* mutant at MS1
GT634-R	CGGTCCATCCCATCCCACAA	Identify *osppr939* mutant at MS1
GP1747-1900-F	GGAGAAAGTCCCCTTGCACT	Identify *osppr939* mutant at MS2
GP1747-1900-R	GGGGTGTGGCACCTAATGAA	Identify *osppr939* mutant at MS2
GP1749-1902-F2	CCGGATAGCCTAACTTACAG	Identify *osppr939* mutant at MS3
GP1749-1902-R2	CTCAATCGTGCTTATACTGC	Identify *osppr939* mutant at MS3
GT635/6-F	GGTGCTGCAAAAACTCGTAA	Identify *osppr939* mutant at MS4, 5
GT635/6-R	CGGTAGGCTGAATACCTTCC	Identify *osppr939* mutant at MS4, 5
SALK_003139LP	CTGCAATAGCAAACAAGCCTC	Identify *tang2* mutant
SALK_003139RP	TGTACGGTGTGTTGATGGATG	Identify *tang2* mutant
LBa1	TGGTTCACGTAGTGGGCCATCG	Identify *tang2* mutant
P1300-939-F	GGCCAGTGCCAAGCTTTACTCTTGGAGATGATCTC	complementation construct
P1300-939-R	GATCGGGGAAATTCGCTAGCACTGACTGTACGTC	complementation construct
OsPPR939N$^{1-280}$-ENF	CACCATGCCTCATGGCTGCCACTT	pGWB5- OsPPR939N$^{1-280}$-GFP
OsPPR939N$^{1-280}$-ENR	CTGCAACGACTCCAATAT	pGWB5- OsPPR939N$^{1-280}$-GFP
PM999- N1-280F	ATCTATCGATTCTAGAATGCCTCATGGCTGCCACTT	pM999- OsPPR939N$^{1-280}$-GFP
PM999- N1-280R	TCACCATGGCTCTAGACTGCAACGACTCCAATATCC	pM999- OsPPR939N$^{1-280}$-GFP
PM999-ΔΔN28F	ATCTATCGATTCTAGAATGGGCGACGGCACCGAGGCG	pM999- OsPPR939N$^{29-280}$-GFP
PM999-ΔΔN28R	TCACCATGGCTCTAGACTGCAACGACTCCAATATCC	pM999- OsPPR939N$^{29-280}$-GFP
S6K1_HAf	CATGGATCCATGGTTTCCTCTGAAA	OsS6K1-HA
S6K1_HAr	ATAGGATCCGCCTAGAGGACTCGGCCT	OsS6K1-HA
PPR939N_GSTf	ATAGGATCCATGCCTCATGGCTGCCAC	939N-GST
PPR939N_GSTr	ATCAGGCCTATCGGCGCGTGGCAGGGA	939N-GST
PPR939-pointM-AF	CTTCCTCCGGCCCCTCGCCTTCACCTGCCG	939S12A-GFP and 939S12A-GST
PPR939-pointM-AR	CGAGGGGCCGGAGGAAGTGGCAGCCATGAG	939S12A-GFP and 939S12A-GST
OsPPR939-probe-F	ATAGCTAAGCAATGGCAC	In situ hybridization probe
OsPPR939-probe-R	CATTAACGCACTGCATAT	In situ hybridization probe
tang2COM-F	GGAGAAAGTCCCCTTGCACT	Identify complemented *tang2*
tang2COM-R	GGGGTGTGGCACCTAATGAA	Identify complemented *tang2*
ppr939COM-F	GGTGCTGCAAAAACTCGTAA	Identify complemented *osppr939-4*
ppr939COM-R	CCAGGCTTTACACTTTATG	Identify complemented *osppr939-4*
Aranad5-F	TTCCCTTTGACCTATGCC	RT-PCR
Aranad5-R	AACTCGGATTCGGCAAGAA	RT-PCR
OsGAPDH-F	CCAAAAGACCGTTGATGGAC	Reference gene in rice
OsGAPDH-R	GAAGAACCTTGCCAACAGCTT	Reference gene in rice
939-qPCR-F	ATGGAAGAGATTTCAAAGCTTTATG	qRT-PCR
939-qPCR-R	CGTTTCCAATTTCCAATCCA	qRT-PCR
AOX1a-F1	CGACAAGATCGCCTACTGG	RT-PCR, qRT-PCR
AOX1a-R1	GGCAGCCATACCTCCTCTG	RT-PCR, qRT-PCR
AOX1b-F	GGAATGGAAGTGGCTCAGT	RT-PCR, qRT-PCR
AOX1b-R	CTCGTGGTGCTTCGTCAC	RT-PCR, qRT-PCR

AOX1c-F1	TGGAAGTGGTCTTGCTTCAGA	RT-PCR, qRT-PCR
AOX1c-R1	GGTCCAGTAGGCGACCTTG	RT-PCR, qRT-PCR
NDA1-F	ATAAGCATCTGGGGAGCATG	qRT-PCR
NDA1-R	AAATACGGCTAATGTCACGG	qRT-PCR
NDA2-F	CTGCTCACCAACCTCATGCT	qRT-PCR
NDA2-R	CTTGACGTAGTCCTTGACGT	qRT-PCR
NDB1-F	GAGTGGTAAAGGTCTCTGAT	qRT-PCR
NDB1-R	GAAGCACAGTCACCAATTGC	qRT-PCR
NDB2-F	AGCAGTTGGAGCTAGGTCTA	qRT-PCR
NDB2-R	CAAAATCGTGCAACTCTGCG	qRT-PCR
NDB3-F	AAGGTACCCACAGGTTGAGT	qRT-PCR
NDB3-R	GTTGAAGCAGTTTGCCAGAT	qRT-PCR
NDC1-F	TGTGGGATTGGGTTACTCTG	qRT-PCR
NDC1-R	GCTACTTGAATCCTCAGATG	qRT-PCR
nad5-F1	CTTTTTGCCTTTGCTCGGTA	RT-PCR
nad5-R1	TATCTGTGCGGATTTCCCAA	RT-PCR
nad5-F2	TTACTCTTCCTGAGTGCGG	RT-PCR
nad5-R2	TACCTAAACCAATCATCA	RT-PCR
nad5-F3	ATATGATGATTGGTTTAGGTA	RT-PCR
nad5-R3	GCAGGAACGATCTGACTAGA	RT-PCR
nad5-F4	GATCAATTCCAACGAGCCTT	RT-PCR
nad5-R4	TCCCACATACGAGAAAAGGT	RT-PCR
atp1-F	TATTGATGGAAAAGGGGCTC	*atp1* abundance
atp1-R	GTCGGCGTAACAATAATGAC	*atp1* abundance
atp6-F	GAGAAGGAGACAAGCAGAAA	*atp6* abundance
atp6-R	GACTTGTCACTGTGAAGCTA	*atp6* abundance
atp9-F	AATAGGTGCCGGAGCTGCTA	*atp9* abundance
atp9-R	AAACAATGCAATAGCTTCGG	*atp9* abundance
ccmB-F	CCAGCCGTCGAAGTGAATGA	*ccmB* abundance
ccmB-R	TCCATGACTTGGCCATTCAA	*ccmB* abundance
ccmC-F	CAGATCTTCAACAAGGTGGA	*ccmC* abundance
ccmC-R	TCGAGCTTCTATTTCTTCCG	*ccmC* abundance
ccmFc-exon1F	CGATAGGTCAGCGAAGCGTG	*ccmFc* abundance
ccmFc-exon2R	AGACCTCGCAAACAACAACGT	*ccmFc* abundance
ccmFn-F	GGCTTTGGGTTATGTAGATC	*ccmFn* abundance
ccmFn-R	GCCTCCTGCTTCATCTGGTA	*ccmFn* abundance
cob-F	CTTTCAACAGCGTAGAACAC	cob abundance
cob-R	CAGAATGTACACCCAATGGA	cob abundance
cox1-F	TGTTTACTGTGGGCTTAGAC	*cox1* abundance
cox1-R	CTGGATAATCTGGAATGCGA	*cox1* abundance
cox2-exon1F	GCTCTGTTATACTCAATGGACGGG	*cox2* abundance
cox2-exon2R	AGATGAGTTTTGGCTGGTACAACC	*cox2* abundance
cox3-F	GAAGGGCATCATACAAAAGC	*cox3* abundance

cox3-R	TATCCGAAATAGTGGAGGGT	*cox3* abundance
matr-F	GTACCCGAATCCATTTACGA	matr abundance
matr-R	AAGAGGAATTTCGTGCTTCT	matr abundance
nad1-exon1F	GCAACGTCGAAAGGGTCCTG	*nad1* abundance
nad1-exon5R	AGGGAGCCATCGAAAGGTGA	*nad1* abundance
nad2-exon1F	CAACCTTCATTTTGCTCATTCATG	*nad2* abundance
nad2-exon5R	TAGAATCCATGTCCTAGGTCTATC	*nad2* abundance
nad3-F	AGATTTAGCGATAAACGGCT	*nad3* abundance
nad3-R	GGCATCCCTCTTTCCTATGT	*nad3* abundance
nad4-exon1F	GGTCCTATTCTCTGTCCCGTGC	*nad4* abundance
nad4-exon4R	CTTACGGATGTATGCATGCAATC	*nad4* abundance
nad4L-F	GGGGAATCCTCCTTAATA	*nad4L* abundance
nad4L-R	CCTCGGACTCGAAAAGTAAT	*nad4L* abundance
nad5-exon1F	CTATAATGACCACCACGTGC	*nad5* abundance
nad5-exon5R	ACCCAAGAAGATAGAGAGTC	*nad5* abundance
nad6-F	TGATGGTTGTACGTGCTAAA	*nad6* abundance
nad6-R	GCATTTCGTCGGAATACATC	*nad6* abundance
nad7-exon1F	AGTGGTGGAACGTGCGGAAC	*nad7* abundance
nad7-exon5R	ACCTTCTGTATAAAGTTCGA	*nad7* abundance
nad9-F	ATTTCCATTGTTGTGCTTTC	*nad9* abundance
nad9-R	CATCATAGCGTACTTCCACA	*nad9* abundance
rpl2-exon1F	ACTTGTTGGAGCTGCTGAGC	*rpl2* abundance
rpl2-exon2R	CCAAGCTCTTGGAACCGAAG	*rpl2* abundance
rpl5-F	TTCATACAGACACAAAGGGG	*rpl5* abundance
rpl5-R	TTTCCCCCTCATCTTTTAGC	*rpl5* abundance
rpl16-F	GGTACACAACTTGGTTTTGG	*rpl16* abundance
rpl16-R	CCACTAACCAATTACGTTACG	*rpl16* abundance
rps1-F	AGCGGATTGAAGAGAGATTC	*rps1* abundance
rps1-R	GATCTGCCGCTATTATCACA	*rps1* abundance
rps2-F	TCCAGAGTTACCAAAACTCG	*rps2* abundance
rps2-R	TGTAGCCAGTCCTTCAAATC	*rps2* abundance
rps3-exon1F	TTTCGGTAAGACTTGATCTGAATCG	*rps3* abundance
rps3-exon2R	TATCCTTTCCGGGTCTTGATTTGTC	*rps3* abundance
rps4-F	GGAAGAGTTGGGTTCGATTC	*rps4* abundance
rps4-R	TTTTCTCCAGATTCTGACCG	*rps4* abundance
rps7-F	CGCATGATCGATGGTAAAAG	*rps7* abundance
rps7-R	CTTTCGGTAAGCATCCAGTA	*rps7* abundance
rps12-F	TAGAGAAGAAAAACAGCGCA	*rpl12* abundance
rps12-R	TTTGGTCTTTCTGCACCATA	*rpl12* abundance
rps13-F	CAAGTCAGAATTGCCTCAAC	*rps13* abundance
rps13-R	TCATTTCCGAATTAGCTTGC	*rps13* abundance
rps19-F	TGGAAGGGAAGTTTTGTTGA	*rps19* abundance
rps19-R	CTGCCCTTTCCTCTATTTGT	*rps19* abundance

orfB-F	GCCTCAACTTGATAAATTGAC	*orfB* abundance
orfB-R	TGGAACATGTGTGAGCATTA	*orfB* abundance
orfX-F	ACCCTTTCTTACCCTACCTT	*orfX* abundance
orfX-R	GTCCAGCCCTCTTCACGAA	*orfX* abundance
orf25-F	GGGATTGAGTTCAACGGATA	*orf25* abundance
orf25-R	AAAGCTTGCACTGTCTTTTC	*orf25* abundance
orf152a-F	AATCCAACCAGAATCCGAAA	*orf152a* abundance
orf152a-R	AGCGGGATAACATTGTCTTT	*orf152a* abundance
orf152b-F	TATGGAGCACTCTTTTCCAC	*orf152b* abundance
orf152b-R	TGCCTTCTCTTTCTGTCTTC	*orf152b* abundance
orf153-F	TTCAAAACAACCACATCAGC	*orf153* abundance
orf153-R	TGCTCACAAGTATCTGTACG	*orf153* abundance
orf161-F	ATAGTACTTCTGACACCGGA	*orf161* abundance
orf161-R	CTGGTTATGTCGCAAAAGAC	*orf161* abundance
orf162-F	ACGGAGAGAATGTTTTACCA	*orf162* abundance
orf162-R	GTAGAACAGAGACACTCGAC	*orf162* abundance
orf165-F	ACTACTCCCCCTTATCCTTC	*orf165* abundance
orf165-R	TAGAGCAAAGGACTGCAAAT	*orf165* abundance
orf176-F	GCAGCGTAATACGATCAAAA	*orf176* abundance
orf176-R	CAACAGGGATGGAAAGGTAT	*orf176* abundance
orf187-F	GGGGAAGGAGTTTCATTCAT	*orf187* abundance
orf187-R	ATCTTCGTCCTACCAAAACC	*orf187* abundance
orf224-F	TTTCTTCAGTGGTTTGTGGA	*orf224* abundance
orf224-R	TTCTCCAAGTTGAGGTTCAG	*orf224* abundance
orf288-F	GACGAGATAATAGGAGGGGA	*orf288* abundance
orf288-R	CAACTTAGCCAAGTCTTCCT	*orf288* abundance
nad1 exon1-2-F	TTGCCATATCTTCGCTAGGTG	nad1 intron 1 splicing
nad1 exon1-2-R	GACCAATAGAGACTTCATAAGGGACCA	nad1 intron 1 splicing
nad1 in1ex2-F	ACGGTTCATAGTTGGGTATG	nad1 intron 1 splicing
nad1 in1ex2-R	GACCAATAGAGACTTCATAAGGGACCA	nad1 intron 1 splicing
nad1 exon2-3-F	CCTTATGAAGTCTCTATTGG	nad1 intron 2 splicing
nad1 exon2-3-R	GACAATCTCACTCGAATTACAG	nad1 intron 2 splicing
nad1 in2ex2-F	CCTTATGAAGTCTCTATTGG	nad1 intron 2 splicing
nad1 in2ex2-R	AGACGTGGACATTCCTCAT	nad1 intron 2 splicing
nad1 exon3-4-F	TGAATCAGTTGCTGGCTATA	nad1 intron 3 splicing
nad1 exon3-4-R	TCATATTGGCATACTCTCC	nad1 intron 3 splicing
nad1 in3ex4-F	ACGTACAGCTCCGTGAGAAGGT	nad1 intron 3 splicing
nad1 in3ex4-R	TCATATTGGCATACTCTCC	nad1 intron 3 splicing
nad1 exon4-5-F	TATGCCAATATGATCTTAATGAGG	nad1 intron 4 splicing
nad1 exon4-5-R	CGAACAAGGGATCTTCTT	nad1 intron 4 splicing
nad1 in4ex4-F	TATGCCAATATGATCTTAATGAGG	nad1 intron 4 splicing
nad1 in4ex4-R	GGTTTATTGCACAAAAAGGC	nad1 intron 4 splicing
nad2 exon1-2-F	ATTATCCACCGTTAGTTAGT	nad2 intron 1 splicing

nad2 exon1-2-R	GGCAATAGTTAGGAGAGG	nad2 intron 1 splicing
nad2 in1ex2-F	AAGAAGTTATCACGGACGAG	nad2 intron 1 splicing
nad2 in1ex2-R	GGCAATAGTTAGGAGAGG	nad2 intron 1 splicing
nad2 exon2-3-F	TGATCTTAGGTGCATTTCC	nad2 intron 2 splicing
nad2 exon2-3-R	TGGCTAATTGATCGAAGTG	nad2 intron 2 splicing
nad2 in2ex3-F	TTTGGAGTCTTTGTGCGAGC	nad2 intron 2 splicing
nad2 in2ex3-R	TGGCTAATTGATCGAAGTG	nad2 intron 2 splicing
nad2 exon3-4-F	AATCACTGGTGCTCAATC	nad2 intron 3 splicing
nad2 exon3-4-R	TATCAGGTGCCCACATAT	nad2 intron 3 splicing
nad2 in3ex4-F	CGAATTAACAACTTGTGGG	nad2 intron 3 splicing
nad2 in3ex4-R	TATCAGGTGCCCACATAT	nad2 intron 3 splicing
nad2 exon4-5-F	GTAGTGACTAGCGTTATAGGTT	nad2 intron 4 splicing
nad2 exon4-5-R	CGATCCATTGGTTCATATAGAATC	nad2 intron 4 splicing
nad2 in4ex5-F	TAGTTAGTACCCCCTTGGTTT	nad2 intron 4 splicing
nad2 in4ex5-R	CGATCCATTGGTTCATATAGAATC	nad2 intron 4 splicing
nad4 exon1-2-F	ATTCTATGTTCTTTCCGAAAGC	nad4 intron 1 splicing
nad4 exon1-2-R	TGTAAATCGGTGGTTCCTGT	nad4 intron 1 splicing
nad4 in1ex1-F	ATTCTATGTTCTTTCCGAAAGC	nad4 intron 1 splicing
nad4 in1ex1-R	CCCTGCAGAGGAAAAGCTT	nad4 intron 1 splicing
nad4 exon2-3-F	TTATACTCTAAGCGCGATTG	nad4 intron 2 splicing
nad4 exon2-3-R	GTCGGTCATATAGAACAC	nad4 intron 2 splicing
nad4 in2ex2-F	TTATACTCTAAGCGCGATTG	nad4 intron 2 splicing
nad4 in2ex2-R	TTGCTAGTAGGTTGATGGGT	nad4 intron 2 splicing
nad4 exon3-4-F	TCCGATCTAAATGGCAGAGAAGT	nad4 intron 3 splicing
nad4 exon3-4-R	GCAATCCGGGAACACTTTGG	nad4 intron 3 splicing
nad4 in3ex3-F	CCGATCTAAATGGCAGAGAAGT	nad4 intron 3 splicing
nad4 in3ex3-R	TACAATGTCATGATCACATTGGT	nad4 intron 3 splicing
nad5 exon1-2-F	CCATGGATCTCATCGGAAAT	nad5 intron 1 splicing
nad5 exon1-2-R	TGGACCAAGCTACTTATGAATG	nad5 intron 1 splicing
nad5 in1ex2-F	TTCGCAGATTGGTCTGACT	nad5 intron 1 splicing
nad5 in1ex2-R	TGGACCAAGCTACTTATGAATG	nad5 intron 1 splicing
nad5 exon2-3-F	CATTCGGGCGAGACAG	nad5 intron 2 splicing
nad5 exon2-3-R	TACCTAAACCAATCATCATAT	nad5 intron 2 splicing
nad5 in2ex2-F	CATTCGGGCGAGACAG	nad5 intron 2 splicing
nad5 in2ex2-R	CGTACGATCGTATCGGGTG	nad5 intron 2 splicing
nad5 exon3-4-F	ATATGATGATTGGTTTAGGTA	nad5 intron 3 splicing
nad5 exon3-4-R	AACTCGGATTCGGCAAGAA	nad5 intron 3 splicing
nad5 in3ex4-F	GCCGTGTAATAGGCGACCA	nad5 intron 3 splicing
nad5 in3ex4-R	AACTCGGATTCGGCAAGAA	nad5 intron 3 splicing
nad5 exon4-5-F	GTTCCTGCGTTTCGGATATT	nad5 intron 4 splicing
nad5 exon4-5-R	AACATTGCAAAGGCATAATGA	nad5 intron 4 splicing
nad5 in4ex5-F	CCTGTAAACCCCCATGATGT	nad5 intron 4 splicing
nad5 in4ex5-R	AACATTGCAAAGGCATAATGA	nad5 intron 4 splicing

nad7 exon1-2-F	GGAACGGGCAAATCAAGAAT	nad7 intron 1 splicing
nad7 exon1-2-R	TTAATTTCTCAGTCCCTCTA	nad7 intron 1 splicing
nad7 in1ex2-F	GATTTGCGAATGAATGCTAG	nad7 intron 1 splicing
nad7 in1ex2-R	TTAATTTCTCAGTCCCTCTA	nad7 intron 1 splicing
nad7 exon2-3-F	AGGGACTGAGAAATTAA TCG	nad7 intron 2 splicing
nad7 exon2-3-R	CTACGGCTGAAGAATGAG	nad7 intron 2 splicing
nad7 in2ex2-F	GTGAACGTGTACGAAAAAGC	nad7 intron 2 splicing
nad7 in2ex2-R	CTACGGCTGAAGAATGAG	nad7 intron 2 splicing
nad7 exon3-4-F	ACGATTAGTGGATATTGG	nad7 intron 3 splicing
nad7 exon3-4-R	GTGCTGCTCTTCGCGAAT	nad7 intron 3 splicing
nad7 in3ex4-F	CACGTACAGTTTGTTTTGGGG	nad7 intron 3 splicing
nad7 in3ex4-R	GTGCTGCTCTTCGCGAAT	nad7 intron 3 splicing
nad7 exon4-5-F	TATGTCCTCCATCACGAT	nad7 intron 4 splicing
nad7 exon4-5-R	AACTGCGGTATAGGTAGA	nad7 intron 4 splicing
nad7 in4ex5-F	TAGCCGGCCAAATGACTAC	nad7 intron 4 splicing
nad7 in4ex5-R	AACTGCGGTATAGGTAGA	nad7 intron 4 splicing
cox2 exon1-2-F	AATGGACGGGGTATTAGTAG	cox2 intron splicing
cox2 exon1-2-R	GTGACTGTTCATCGGAACTG	cox2 intron splicing
cox2 in1ex1-F	AATGGACGGGGTATTAGTAG	cox2 intron splicing
cox2 in1ex1-R	TTCGTTGCACTTAAATCACCC	cox2 intron splicing
rps3 exon1-2-F	CGGTAAGACTTGATCTGAAT	rps3 intron splicing
rps3 exon1-2-R	AAGGTGAGTATCGTAGGT	rps3 intron splicing
rps3 in1ex1-F	CGGTAAGACTTGATCTGAAT	rps3 intron splicing
rps3 in1ex1-R	TCCTCTCACGCCTTTTTTTG	rps3 intron splicing
rpl2 exon1-2-F	GAAGGATGGAGCGTACAA	rpl2 intron splicing
rpl2 exon1-2-R	TGGAGCAATCGCAATTTATC	rpl2 intron splicing
rpl2 in1ex1-F	GAAGGATGGAGCGTACAA	rpl2 intron splicing
rpl2 in1ex1-R	CTGTTTCTAGGTGGGTCG	rpl2 intron splicing
ccmFc exon1-2-F	CACATGGAGGAGTGTGCATC	ccmFc intron splicing
ccmFc exon1-2-R	GTGGGTCCATGTAAATGATCG	ccmFc intron splicing
ccmFc in1ex1-F	CACATGGAGGAGTGTGCATC	ccmFc intron splicing
ccmFc in1ex1-R	CCCGGATCGAATCAGAGTT	ccmFc intron splicing
